# Analysis of Several Pathways for Efficient Killing of Prostate Cancer Stem Cells: A Central Role of NF-κB RELA

**DOI:** 10.3390/ijms22168901

**Published:** 2021-08-18

**Authors:** Kaya E. Witte, Jesco Pfitzenmaier, Jonathan Storm, Melanie Lütkemeyer, Clara Wimmer, Wiebke Schulten, Nele Czaniera, Marvin Geisler, Christine Förster, Ludwig Wilkens, Cornelius Knabbe, Fritz Mertzlufft, Barbara Kaltschmidt, Jan Schulte am Esch, Christian Kaltschmidt

**Affiliations:** 1 Department of Cell Biology, University of Bielefeld, Universitätsstrasse 25, 33615 Bielefeld, Germany; jonathan.storm@uni-bielefeld.de (J.S.); Melanie-luetkemeyer@gmx.de (M.L.); Clarawimmer@gmx.de (C.W.); Wiebke-schulten@web.de (W.S.); Nele.cz@web.de (N.C.); Marvin-geisler@web.de (M.G.); Barbara.Kaltschmidt@uni-bielefeld.de (B.K.); 2 Forschungsverbund BioMedizin Bielefeld, OWL (FBMB e.V.), Maraweg 21, 33617 Bielefeld, Germany; Jesco.Pfitzenmaier@evkb.de (J.P.); christine.foerster@krh.eu (C.F.); ludwig.wilkens@krh.eu (L.W.); cknabbe@hdz-nrw.de (C.K.); fritz.mertzlufft@evkb.de (F.M.); Jan.SchulteamEsch@evkb.de (J.S.a.E.); 3 Department of Urology and Center for Computer-Assisted and Robotic Urology, Protestant Hospital of Bethel Foundation, Campus Bielefeld-Bethel, University Medical School OWL at Bielefeld, Bielefeld University, Burgsteig 13, 33617 Bielefeld, Germany; 4 Institute of Pathology, KRH Hospital Nordstadt, Affiliated with the Protestant Hospital of Bethel Foundation, 30167 Hannover, Germany; 5 Heart and Diabetes Centre NRW, Institute for Laboratory and Transfusion Medicine, Ruhr-University Bochum, 32545 Bad Oeynhausen, Germany; 6 Protestant Hospital of Bethel Foundation, Campus Bielefeld-Bethel, University Medical School OWL at Bielefeld, Bielefeld University, Maraweg 21, 33617 Bielefeld, Germany; 7 Molecular Neurobiology, Faculty of Biology, Bielefeld University, Universitätsstrasse 25, 33615 Bielefeld, Germany; 8 Department of General and Visceral Surgery, Protestant Hospital of Bethel Foundation, Campus Bielefeld-Bethel, University Medical School OWL at Bielefeld, Bielefeld University, Burgsteig 13, 33617 Bielefeld, Germany

**Keywords:** prostate cancer, cancer stem cells, nuclear factor “kappa-light-chain-enhancer” of activated B-cells, tumor necrosis factor, interferon gamma, immune checkpoint therapy, programmed cell death 1 ligand 1 and -ligand 2, natural killer cells, major histocompatibility complex class I

## Abstract

Prostate cancer is a common cause of death worldwide. Here, we isolated cancer stem cells (CSCs) from four adenocarcinomas of the prostate (Gleason scores from 3 + 3 up to 4 + 5). CSCs were characterized by the expression of the stem cell markers *TWIST*, the epithelial cell adhesion molecule (*EPCAM*), the transcription factors SNAI1 (*SNAIL*) and SNAI2 (*SLUG*) and cancer markers such as CD44 and prominin-1 (CD133). All investigated CSC populations contained a fraction highly positive for aldehyde dehydrogenase (ALDH) function and displayed robust expressions of programmed cell death 1 (PD-1) ligands. Furthermore, we investigated immunotherapeutic approaches but had no success even with the clinically used PD-1 inhibitor pembrolizumab. In addition, we studied another death-inducing pathway via interferon gamma signaling and detected high-level upregulations of human leukocyte antigen A (HLA-A) and beta 2-microglobulin (B2M) with only moderate killing efficacy. To examine further killing mechanisms in prostate cancer stem cells (PCSCs), we analyzed NF-κB signaling. Surprisingly, two patient-specific populations of PCSCs were found: one with canonical NF-κB signaling and another one with blunted NF-κB activation, which can be efficiently killed by tumor necrosis factor (TNF). Thus, culturing of PCSCs and analysis of respective NF-κB induction potency after surgery might be a powerful tool for optimizing patient-specific treatment options, such as the use of TNF-inducing chemotherapeutics and/or NF-κB inhibitors.

## 1. Introduction

Prostate cancer (PCa) is the fourth most common cancer worldwide and the most frequent non-cutaneous cancer in men, leading to an estimated 375,000 deaths per year [1]. Due to a lack of pathological symptoms before diagnosis [2], in most cases, PCa remains incurable after forming metastases [3]. Most PCa patients exhibit increased plasmatic amounts of prostate-specific antigen (PSA). The screening of PSA in clinical practice, however, may result in over-diagnosis to some extent [4,5]. In low-risk PCa patients, an active surveillance strategy has turned out to be the most popular option, avoiding therapy that results in side effects and overtreatment [6]. In intermediate and high-risk PCa, curative treatment is favorable, with common treatment options being radiation therapy, androgen-ablation, and radical prostatectomy [7,8]. Alternatively, high-intensity focused ultrasound or cryotherapy may be chosen, although these are mainly used in low- or intermediate-risk PCa [9,10]. Prior to treatment, individual PCa stages are defined, with prostatic adenocarcinomas being the predominant variant [3]. To precisely assess cancer aggressiveness and thus determine appropriate therapy options, the Gleason score or the resulting WHO grading systems are recommended [11,12]. Both systems are based on the evaluation of histological tumor structures, differentiation stages, and gland cell morphologies. In our study, four donated PCa samples were identified as acinar adenocarcinomas with Gleason scores from 3 + 3 to 4 + 5. They were used to isolate the specific prostate cancer stem cells (PCSCs), which are commonly described to be responsible for PCa formation, progression, and metastatic formation [13]. The molecular characterization of PCSCs might allow for improvements in patient-specific PCa therapy.

PCa progression is strongly related to androgen signaling through androgen receptor (AR)-regulated gene expression. However, in PCa, the AR is frequently amplified, mutated, or exhibits polymorphisms causing severe tumor growth [14,15]. Due to the high proliferative responsiveness of prostatic carcinomas to hormones, the ablation of androgens serves as a standard therapy after PCa diagnosis [16]. Another key mechanism in cancer is epithelial-to-mesenchymal transition (EMT), which is linked to metastatic progression in a wide range of different cancers, including PCa [17]. EMT enables PCSCs to enter the bloodstream and results in metastatic formation in distinct tissues, such as bone or lymph node metastases [18,19]. PCa metastases in bone have been reported to interact with the surrounding bone microenvironment, leading to bone destruction and promoting cancer cell survival and tumor progression, which in turn results in poor prognosis for PCa patients [19]. Collin and co-workers firstly isolated PCSCs, expressing the now well-established CSC markers CD44 and prominin-1 (CD133) [20,21]. In recent literature, further markers, such as aldehyde dehydrogenase 1 (ALDH1) and Nestin, have also been described on PCSCs [22,23,24]. Moreover, the expression of EMT-associated genes, such as the epithelial cell adhesion molecule (*EPCAM*) as well as the transcription factors SNAI1 (*SNAIL*), SNAI2 (*SLUG*) and *TWIST*, are commonly known to be related to CSCs [25,26,27,28]. Additionally, the transcription factor and proto-oncogene *MYC* plays an essential role in cancer and tumor progression. We recently published transcriptomic data on four different cancer types, wherein 11 out of 12 individual CSC populations showed *MYC* expression and thus emphasizes the overall presence of MYC in CSCs [29]. Furthermore, the importance of MYC in context with PCa cancer initiation, metastasis, and the stimulation of embryonic stem cell-like signatures in chemoresistant cancer cells was described and reviewed [30]. Since common treatments, such as chemotherapeutic agents, do not specifically affect CSCs but may affect the patient in other ways, an increasing number of therapeutic strategies focus on immunotherapy. In this context, different genes, such as the programmed cell death 1 ligands 1 and 2 (PD-L1/-2), play an important role in the evasion of the immune system [31]. On the mechanistic level, CSC-associated proteins like PD-L1 and -L2, induced through pro-inflammatory cytokines such as tumor necrosis factor alpha (TNFα) or interferon gamma (IFNγ), bind to the checkpoint receptor programmed cell death 1 (PD-1) and lead to impaired immune cell proliferation [32,33,34,35,36,37]. In particular, PD-1 is commonly represented on various immune cells, such as T-cells, B-cells or natural killer (NK) cells [32,33]. While B-cells function mainly in antibody-mediated immune reactions, T-cells are commonly responsible for recognizing specific molecular patterns presented on the major histocompatibility complex (MHC) class I molecules. NK cell-mediated cytotoxicity is regulated by a plethora of activating and inhibiting receptors within a complex network of signal integration, including the inhibition of respective cytotoxic functions through specific binding of MHC class I molecules [38,39,40]. In addition to blocking the interaction between immune checkpoint receptors and ligands, the induction of the systemic innate immune response against cancer-associated antigens is another course of PCa immunotherapy [41,42,43]. A recent review described the decisive role of NK cells within the elimination of cancer cells [44]. However, CSCs from PCa patients can escape NK cell-mediated lysis via different mechanisms [45,46]. Therefore, treatment strategies based on targeting cancer cells expressing the previously mentioned MHC class I molecules are the focus of current research. The human leukocyte antigen (HLA) system, specifically MHC class I, is encoded by the *HLA-A*, *-B*, and *-C* genes, as well as by beta 2-microglobulin (*B2M*), and plays a central role in the tumor mechanisms of evading the patient’s immune system [47,48]. Thus, elevated B2M expression in patients with advanced and localized PCa in contrast to healthy prostate B2M levels [49] justifies the focus on HLA expression in cancer cells as one strategy to prevent immune surveillance. Consequently, deriving detailed mechanistic insights and precisely targeting PCa cells with stemness characteristics represent a promising approach to PCa treatment.

Emphasizing the molecular understanding of tumor progression and respective cellular treatment mechanisms, the nuclear factor “kappa-light-chain-enhancer” of activated B-cells (NF-κB) is widely described as the major transcription factor of inflammation and cell survival. NF-κB is a multi-subunit transcription factor family consisting of DNA-binding subunits, such as p50, p52, and the REL oncogene homology domain proteins RELA, cREL and RELB. NF-κB deregulation is strongly associated with the progression and formation of tumors [50,51]. In detail, the activation of NF-κB initiates the transcription of genes related to tumorigenesis, cellular survival, proliferation and invasive potential, as well as to angiogenesis [52]. During the progression of solid cancers, an inflammatory microenvironment is formed by the recruitment of tumor-infiltrating immune cells, resulting in tumoral growth and cytokine secretion [53]. The presence of cytokines such as TNFα, in turn, activates canonical NF-κB signaling pathways in solid cancers, enhancing the expression of genes associated with chemoresistance and metastasis [53,54,55]. Consequently, many CSC-associated genes are known to activate NF-κB such as PD-L1 and −2, or are reported to be correlated with specific transcription factors’ activation, such as SNAIL [34,35,36,37,56,57]. Regarding immunotherapy, we further addressed the NF-κB-mediated expression of *HLA-A* and *-B* in our recent study of transcriptomic CSC analysis [29]. Thus, detailed analyses of the NF-κB pathways’ potential involvement in killing PCSCs might be of particular interest for future patient-specific PCa therapies. 

In the present study, we established primary PCSC populations of four parental PCa patients and analyzed several pathways known for the killing of CSCs. Firstly, we analyzed NK cell-mediated immunotherapy and observed elevated levels of PD-L1/-2 expression. Additionally, no effective cancer cell lysis was achieved by co-culturing with the PD-1 inhibitor pembrolizumab. Secondly, cellular treatment with the pro-inflammatory cytokine IFNγ resulted in the high-level upregulation of the MHC class I molecules B2M and HLA-A2, but killing was only moderately induced by IFNγ treatment. Thirdly and unexpectedly, the analysis of NF-κB signaling in PCSCs showed dual effects. As expected, in two PCSC cultures, TNFα treatment led to canonical NF-κB activation and cell survival. Contrarily, in two other populations, TNFα treatment resulted in the efficient killing of PCSCs, but without NF-κB activation. Together, our findings provide strong evidence of the central role of NF-κB RELA signaling in PCSCs. 

## 2. Results

### 2.1. Primary Isolated Prostate Cancer Cells Exhibit Stemness Characteristics

**Figure 1:**
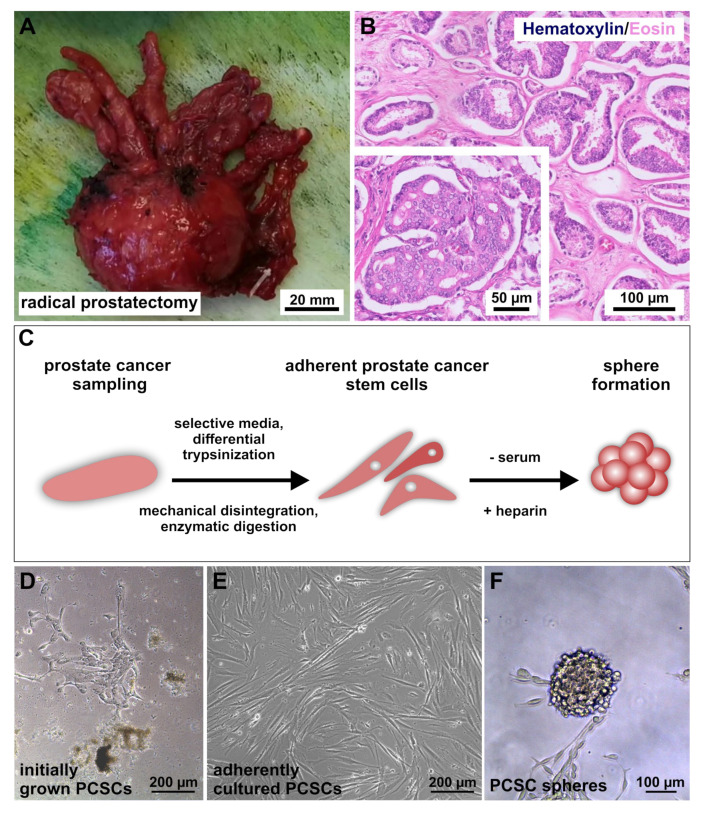
Isolation and cultivation of primary putative prostate cancer stem cells. (**A**) Photograph of a cancerogenic prostate (for sample PIX) after a radical robotic-assisted prostatectomy. (**B**) Exemplary hematoxylin/eosin stainings from PXI acinar adenocarcinoma with a Gleason score of 3 + 4. The dominant pattern 3 (outer segment) depicts variably sized individual glands, while the inner segment further shows minor pattern 4 with cribriform glands. (**C**) Schematic procedure after surgical removal of tumor-infiltrated prostates. Tissue samples were minced, digested and further cultured under cancer stem cell-selecting conditions after enrichment via differential trypsinization processes. (**D**–**F**) Successfully cultured putative prostate cancer stem cells (PCSCs, example from donor PXI) after isolation from primary tumor tissues. Cells grew adherently by cultivation with fetal calf serum (**D**,**E**) and were able to form spheres via the replacement of serum with heparin (**F**).

In this study, four patients aged between 57 and 83 were included who had been previously diagnosed with PCa. Patient 3 (donor of PXIII PCSC population) possessed a combination of muscle-invasive bladder cancer (BCa) and PCa. Each patient underwent a radical prostatectomy (RPx; exemplary depicted in Figure 1A), whereas patient 3 underwent a radical cystoprostatectomy (CPx) (cf. Table 1). To achieve precise grading through Gleason scores, hematoxylin/eosin staining was performed (exemplary images in Figure 1B) and tumor tissue samples for PCSC isolation were obtained. Histopathology revealed acinar adenocarcinomas in all patients, and PXIV’s tumor displayed a locally advanced stage of the respective carcinoma. Tumor typing and the specific characterization of PCa donors are listed in Table 1.

For the isolation and cultivation of primary PCSCs, the received tumor tissues were minced, digested, adherently cultured in selective CSC media and enriched via differential trypsinization processes, as recently described [29] (Figure 1C). Chemically defined CSC media was supplemented with epidermal growth factor (EGF) and basic fibroblast growth factor 2 (bFGF-2), as well as with 10% fetal calf serum (FCS), to obtain trypsin-sensitive PCSC populations with low attachment capabilities. After serial trypsin treatment, adherently grown PCSCs developed typical spindle- and mesenchymal-shaped morphologies (Figure 1D,E). For further culturing as free-floating spheres, the primary cancer cells were cultivated with heparin and in the absence of FCS (Figure 1E and Figure S1). Three of the four PCSC populations grew as spheres (see Figure 1 and Figure S1A), while PXIV PCSCs, which possessed the highest Gleason grading, also grew adherently under serum-free conditions with heparin.

**Figure 2:**
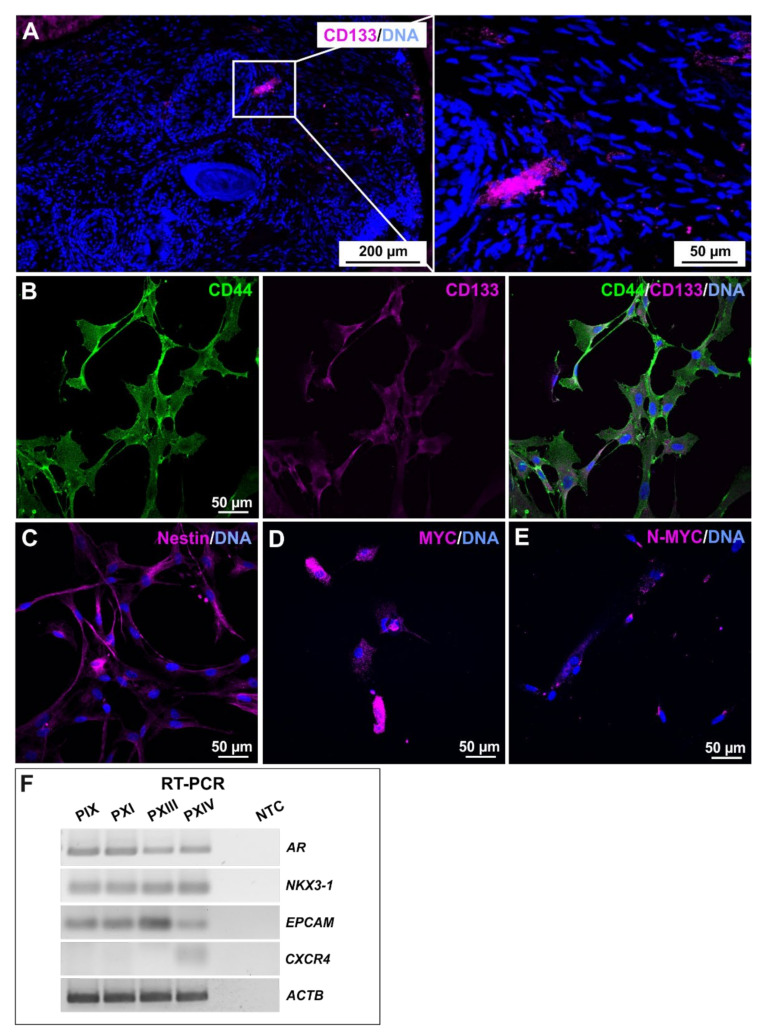
Characterization of stemness properties in primary prostate cancer stem cells. (**A**) Identification of prominin-1 (CD133)-positive cells, localized as a small group of clustered cells after immunohistochemistry of an exemplary prostate cancer section (donor PXI). (**B**,**C**) Immunocytochemical stainings of cancer stem cell (CSC) markers CD44, CD133 and Nestin in the cultured putative prostate cancer stem cell (PCSC) population of PXI. The co-expression of CD44/CD133 revealed the stemness character of primary isolated PCSCs (**B**), further verified by the presence of Nestin (**C**). (**D**,**E**) Exemplary proto-oncogene MYC and MYC family member N-MYC immunocytochemistry analyses of PIX PCSCs depicted expressions of both CSC-associated markers at the protein level. Nuclear counterstaining was performed using DAPI. (**F**) The further detection of prostate cancer and PCSC markers were carried out on the mRNA level after reverse-transcription (RT) PCR, whereas the androgen receptor (*AR*), prostate cancer-specific gene *NKX3-1*, and CSC-related gene epithelial cell adhesion molecule (*EPCAM*) were detected in each PCSC population, the metastasis-associated gene *CXCR4* was exclusively detected in PXIV PCSCs. Beta actin *(ACTB)* served as the housekeeping gene.

Verification of the stemness in primary isolated PCa cell populations was conducted by analyzing the specific expression profiles at the mRNA and protein levels (Figure 2). A paraffin-embedded section of cancerogenic prostate tissue (donor PXI) was exemplarily stained immunohistochemically for the well-established CSC marker CD133 (Figure 2A). The CD133-positive cells were arranged in a small nest and were not individually present in the examined PCa tissue. The CD44/CD133 co-expression of adherently cultured cells, as well as the presence of Nestin, MYC and MYC family member N-MYC, confirmed the stemness of the isolated PCSC populations (Figure 2B–E, Figures S1 and S2). Reverse-transcription (RT) PCR amplified *AR,* PCa-specific homeobox gene *NKX3-1* and *EPCAM* transcripts for each PCSC population, each within similar ranges, with the exception of *EPCAM*, the expression of which was elevated in PXIII PCSCs, which possessed the lowest Gleason score of 3 + 3 (Figure 2F and Figure S3). Interestingly, only PXIV PCSCs expressed *CXCR4*, which, considering the tumor grading at the time of surgery, might indicate potential for a future metastatic progression [58]. 

### 2.2. Prostate Cancer Stem Cells Display High Levels of Epithelial-to-Mesenchymal Transition-Associated Genes and Distinct Expressions of Programmed Cell Death Ligands

**Figure 3:**
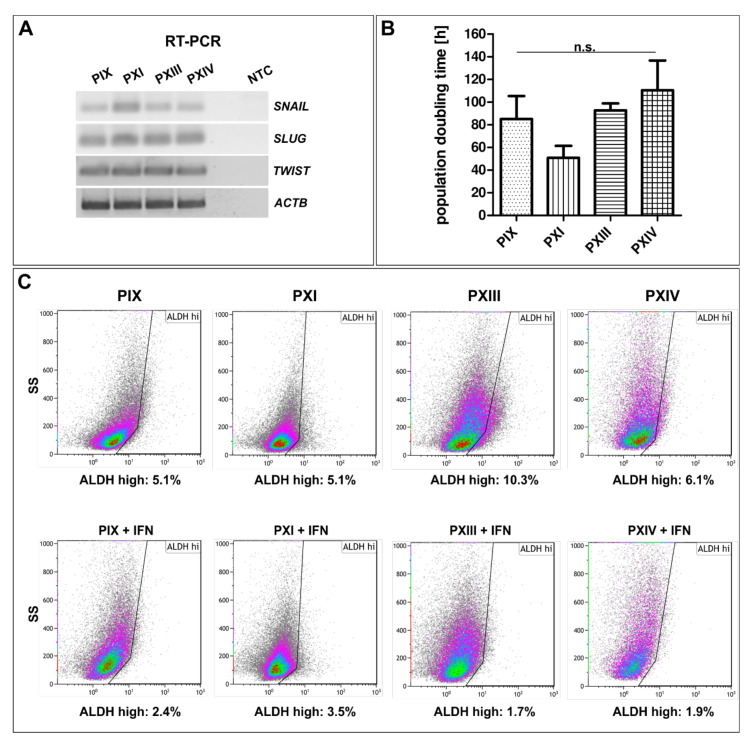
Gene expressions associated with epithelial-to-mesenchymal transition, proliferative behavior and aldehyde dehydrogenase activity analysis. (**A**) Target gene amplifications analyzed by reverse-transcription (RT) PCR of the epithelial-to-mesenchymal transition (EMT)-associated transcription factors SNAI1 (*SNAIL*), SNAI2 (*SLUG*) and *TWIST* in primary prostate cancer stem cells (PCSCs). Beta actin (*ACTB*) served as a housekeeping gene. (**B**) PCSC proliferation analyses showed population doubling times between 50 h (PXI) and 100 h (PXIV), which were not significantly different following Mann–Whitney U tests (for each population *n* = 3). Means are displayed as the standard error of the mean (SEM). (**C**) Flow cytometry of aldehyde dehydrogenase (ALDH) activities in PCSCs showed that up to 10% of cells had high ALDH protein expressions (PXIII, upper panel). These were reduced to less than 4% in the examined PCSC populations after pre-treatment with interferon gamma (lower panel).

After to the expressions of PCa-associated and CSC markers, the cancer-specific gene profiles of isolated PCSCs and their respective proliferation rates were investigated in detail (Figure 3). Here, all analyzed PCSC populations showed gene expressions of *SLUG*, *SNAIL* and *TWIST* (Figure 3A and Figure S3), verifying the EMT characteristics of the mesenchymal-shaped PCSCs in this study. SNAIL expression, which is directly correlated with the NF-κB transcription factor [57], seems to be higher in PXI PCSCs, with its parental tumor tissue being graded with a Gleason score of 3 + 4. 

As another characteristic, the specific proliferation rates of cultured PCSC populations were determined and compared (Figure 3B). The evaluated population doubling times differed from around 50 h for PXI PCSCs to 100 h for PXIV, and between 80 h and 90 h for PIX and PXIII. However, no significant discrepancies could be detected during the statistical analysis, suggesting a slowly dividing cell cycle and the subsequently decelerated DNA replication in the primary isolated PCSC populations.

As briefly mentioned above, cancer cells expressing ALDH potentially promote chemoresistance, and *ALDH1* is present as a marker gene of PCSCs [24]. Therefore, ALDH activities were measured via flow cytometry, including a partial pre-treatment with the pro-inflammatory cytokine IFNγ (Figure 3C and Figure S4 for DEAB controls). Here, each population presented as a small subgroup with high ALDH activity (upper panels, maximum 10%, see PXIII PCSCs), which was reduced via pre-treatment with IFNγ (lower panels), indicating a correlation between the cytokine-mediated pathway and the activity of a chemoresistance-associated protein.

**Figure 4:**
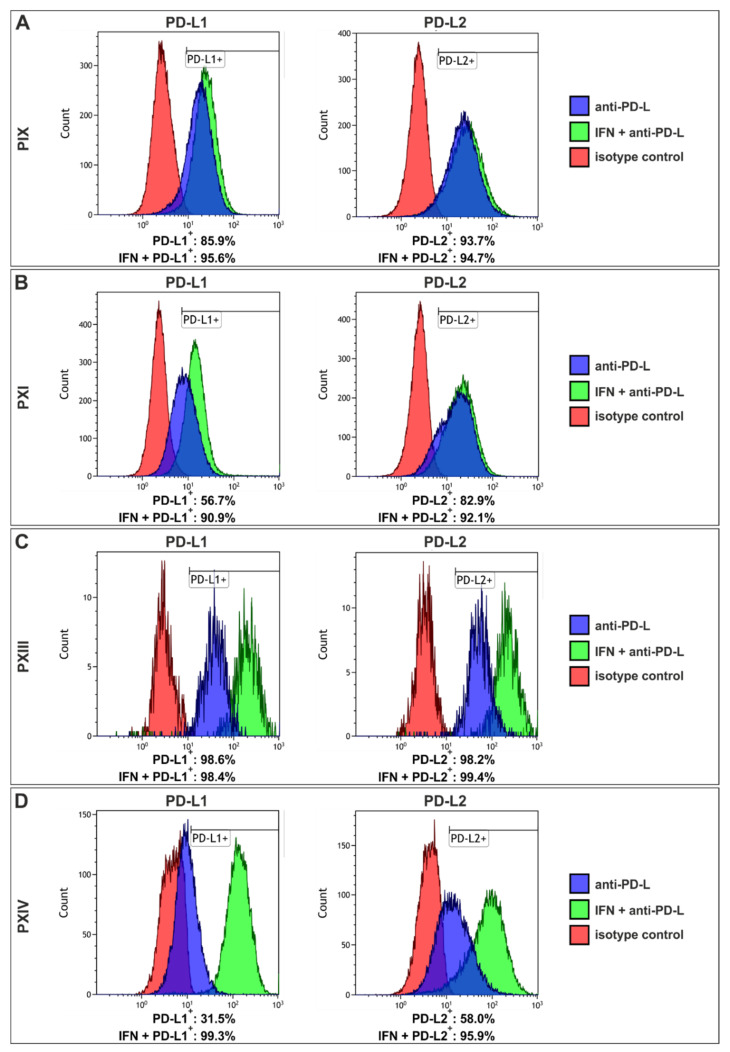
Prostate cancer stem cells showed enhanced affinity to programmed cell death 1 after interferon treatment. (**A**) Flow cytometry of the PIX prostate cancer stem cell (PCSC) population depicted increased expressions of the programmed cell death 1 ligand 1 (PD-L1; left panel) after 72 h of incubation with interferon gamma (IFNγ; green graph), in comparison with untreated PCSCs (blue graph). Analyses of PD-L2 expressions (right panel) showed no effect of IFNγ pre-treatment. (**B**) PXI PCSCs also displayed a strong increase in PD-L1^+^ cells after incubation with IFNγ (left panel), as compared to the expression of PD-L1 alone. The analysis of PD-L2 showed a difference of 9% in positive cells between pre- and untreated PCSCs. (**C**) PXIII PCSCs contained 98% of both PD-L positive stained cells, independent of IFNγ. (**D**) The PCSCs population PXIV revealed the highest increase in PD-L^+^ cells after treatment with IFNγ, up to 67% (PD-L1). For all analyses, red graphs display the isotype controls.

Since PD-L1 and -L2 were previously reported as crucial CSC-associated proteins [32,33], the expression of PD-L1 and -L2 was examined via flow cytometry (Figure 4 and Figure S5 and Table S1), again with (green graphs) and without (blue graphs) an IFNγ pre-treatment. The pre-treatment with IFNγ resulted in increased counts of PD-L1-positive cells in PIX (86% to 96%), PXI (57% to 91%) and PXIV (32% to 99%) (Figure 4A,B,D, left panel), as well as increased PD-L2^+^ cell counts in PXI (83% to 92%) and PXIV (58% to 96%) (Figure 4B,D, right panel). PXIII’s PCSC contained more than 98% PD-L1/2^+^ independently of IFNγ (Figure 4C), and PIX contained more than 93% PD-L2-positive cells without previous cytokine treatment (Figure 4A, right panel). Of note, the PXIV PCSCs (Gleason score 4 + 5) displayed the greatest increase in PD-L positive cells after IFNγ treatment, as contrary to PXIII PCSCs (Gleason Score 3 + 3), which showed no enrichment in PD-L1/2^+^ cells after culturing with IFNγ. However, the PCSC populations PXIII and PXIV showed a more significant increase in PD-L expression levels than PIX and PXI, as depicted by the shift in fluorescence intensity after treatment with IFNγ (see Table S1).

### 2.3. Programmed Cell Death 1 Inhibitor Pembrolizumab Caused No Enhancement of Low-Level Natural Killer Cell-Mediated Lysis on Primary Prostate Cancer Stem Cells

As explained above, the innate immune system plays a central role in various anti-tumor strategies. NK cells, for example, specifically target CSCs [44]; however, different cancer-associated mechanisms inhibit NK cell-mediated tumor cell lysis, especially in PCSCs [59]. Therefore, we analyzed the effects of NK cell-based cytotoxicity on PCSCs pre-treated with IFNγ after a partial co-culture with the PD-1 inhibitor pembrolizumab, given that an effective enhancement of NK cell-mediated cytotoxicity has previously been reported following its application [60].

**Figure 5:**
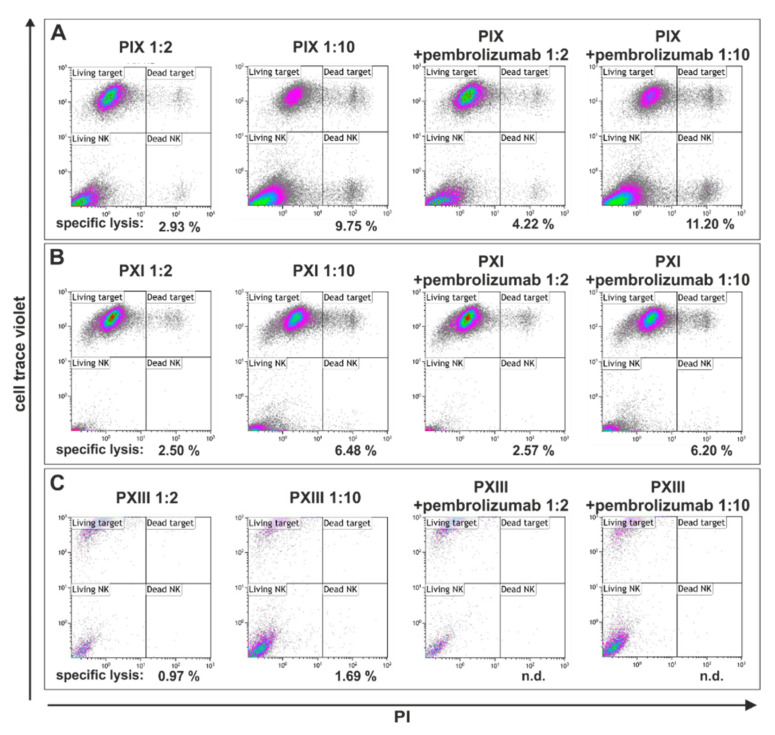
Flow cytometry of natural killer cell-treated prostate cancer stem cell populations showed no effect on cellular survival even with the addition of pembrolizumab. (**A**) The analysis of the natural killer (NK) cell-treated prostate cancer stem cell (PCSC) population PIX showed a killing efficacy of only about 11%, also with pembrolizumab co-culturing (1 target: 10 NK cells; right panels). Without pembrolizumab, the killing rates differed from 2.9% (1 target: 2 NK cells) to 9.8% (1 target: 10 NK cells; left panels). (**B**) The NK cell-mediated lysis of PXI PCSCs showed killing efficacies in a similar range to PIX PCSCs. Flow cytometry revealed a target lysis of 2.5% (1 target: 2 NK cells) to 6.5% (1 target: 10 NK cells) and a maximum killing efficacy of 6.2% with the addition of pembrolizumab. (**C**) PCSCs from PXIII suffered the lowest cytotoxic effects from the NK cell co-culture, while treatment alone led to a maximum specific lysis of 1.7%. The combination with pembrolizumab revealed no detectable NK cell killing.

On the mechanistic level, pembrolizumab binds the PD-1 receptor on NK cells, preventing the binding of the ligands PD-L1/PD-L2 expressed on cancer cells, and thus excluding this cancer-related survival strategy. Here, three PCSC populations from the parental tumor tissues PIX, PXI and PXIII were treated and subsequently analyzed via flow cytometry (Figure 5). Co-culturing for 4 h in two different ratios of PCSCs: NK cells (1:2 and 1:10) revealed a specific lysis of only 11.2%, which was also detected in PIX PCSCs (ratio 1:10) after the addition of pembrolizumab (Figure 5A, right panel). The treatment of PXI PCSCs also led to a maximum of 6.5% dead target cells with no PD-1 inhibitor (Figure 5B, left panel). The PCSC population PXIII showed nearly no cytotoxic effects, with a maximum of only 2% specific lysis without the addition of pembrolizumab and no measurable lysis in the presence of pembrolizumab (Figure 5C). Consequently, our findings suggest no significant effect of NK cell-mediated cytotoxicity on primary isolated PCSCs, even in the presence of the PD-1 inhibitor pembrolizumab.

### 2.4. Interferon Gamma Treatment of Prostate Cancer Stem Cells Revealed High-Level Upregulation of Major Histocompatibility Complex Class I with Only Moderate Cytotoxic Effects

**Figure 6:**
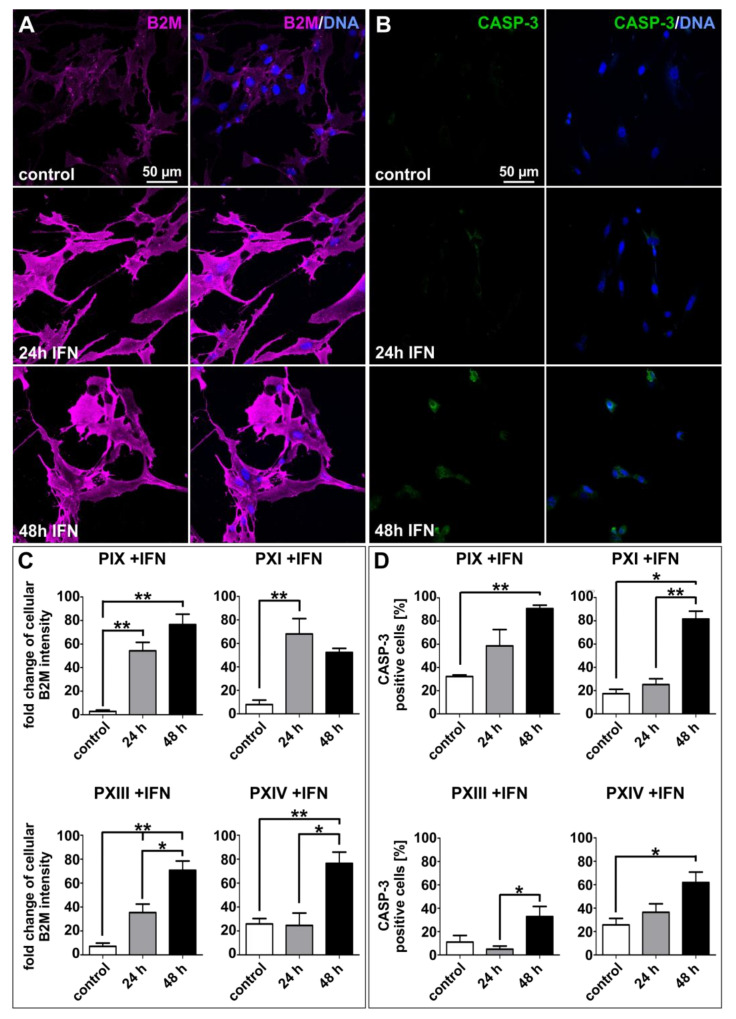
Interferon gamma treatment upregulated beta 2-microglobulin while only slightly inducing cleaved Caspase-3 in primary prostate cancer stem cells. (**A**,**B**) Exemplary immunocytochemical beta 2-microglobulin (B2M, **A**) and apoptosis-related marker cleaved Caspase-3 (CASP-3, **B**) stainings of control and the 24 or 48 h interferon gamma (IFNγ)-treated PXI PCSC population. (**C**,**D**) The effects of B2M immunostainings were statistically significant (**C**) but displayed only moderate IFNγ-related inductions of CASP-3 protein (**D**). Image quantification was performed using ImageJ software (NIH, Bethesda, MD, USA). Statistical analysis was performed with Mann–Whitney U tests (minimum 50 cells per analyzed protein, condition and population), * *p* ≤ 0.05 and ** *p* ≤ 0.01 were considered significant. Means are displayed as standard error of the mean (SEM).

IFNγ secretion activates various signaling pathways involved in immune-based mechanisms, such as the previously mentioned regulation of the checkpoint receptor ligands PD-L1/-L2 (see Figure 4) [34,37], which in turn can lead to the prevention of NK cell-mediated lysis in PCSCs. In this context, current research focuses on the adaptive immune system, with MHC class I molecules playing a central role. In line with a previously mentioned study in which PCa patients showed enhanced B2M levels compared to normal [49], we immunocytochemically analyzed MHC class I expression via B2M staining, and the respective cellular state via the common apoptosis marker cleaved Caspase-3 (CASP-3) in primary PCSCs after 24 and 48 h of IFNγ treatment (Figure 6A,B, Figures S7 and S8). The expressions of B2M and CASP-3 were enhanced during treatment with IFNγ, and statistical analyses (Figure 6C,D) revealed significant B2M upregulations in each investigated PCSC population compared to untreated controls (Figure 6C). Interestingly, the amount of CASP-3-positive cells during cytokine treatment was also statistically relevant in the analyzed PCSCs (Figure 6D). However, the examined CASP-3 protein expressions remained at a moderate level, since the immunocytochemical staining depicted only gentle expressions, even after 48 h of IFNγ treatment (see also Figure S8). 

**Figure 7:**
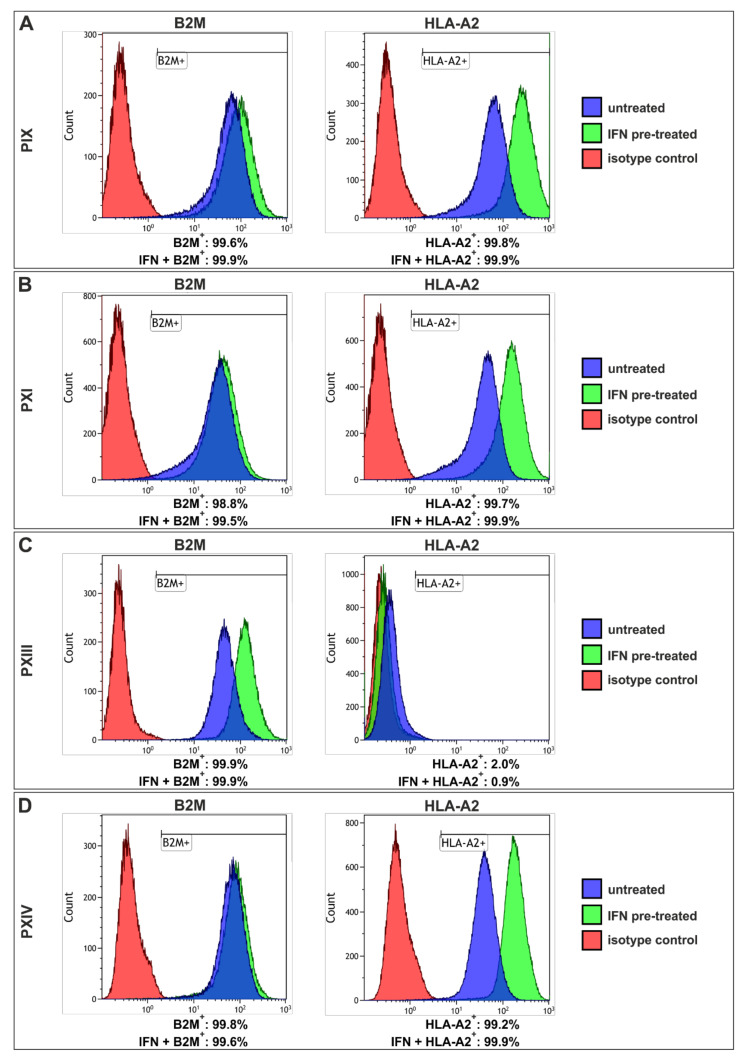
Flow cytometry revealed high-level expressions of beta 2-microglobulin and human leukocyte antigen A2 in three prostate cancer stem cell populations. (**A**–**D**) Analyses showed over 98% beta 2-microglobulin (B2M)^+^ cells in prostate cancer stem cells (PCSCs) (left panel, blue graphs), similar to interferon gamma (IFNγ) pre-treated cells (green graphs). The human leukocyte antigen A2 (HLA-A2) staining (right panels) in PIX, PXI and PXIV PCSCs revealed more than 99% positive cells (**A**,**B**,**D**). The PCSC population PXIII displayed a maximum of 2% HLA-A2^+^ cells (**C**, right panel) when untreated (blue graph), and only 0.9% after treatment with IFNγ (green graph). For all the analyses, isotype controls are displayed as red graphs.

Flow cytometric analysis of the MHC light chain (B2M) and heavy chain (for example, HLA-A2) (Figure 7, Figures S9 and S10 and Table S2) revealed the presence of more than 99% B2M^+^ cells in each PCSC population, independent of IFNγ (Figure 7A–D, left panels), while the B2M expression levels further rose in response to IFNγ (see Table S2). Moreover, PIX, PXI and PXIV PCSCs exhibited over 99% HLA-A2-positive cells (Figure 7A,B,D, right panel) without IFNγ pre-treatment (blue graphs), and a clear shift towards higher HLA-A2 expression levels could also be observed upon IFNγ pre-treatment in these populations (see Table S2). Surprisingly, PXIII PCSCs (Figure 7C) showed nearly no HLA-A2^+^ signals with or without IFNγ treatment (green graph); however, the B2M expression level (light chain of MHC class I) indicated the presence of other heavy chain MHC class I members. As such, the natural MHC class I expression level in isolated PCSCs could be enhanced by treatment with the pro-inflammatory cytokine IFNγ. CASP-3 expression could be induced simultaneously, but here it was here kept at a moderate level, suggesting no pronounced PCSC killing efficacy was induced by IFNγ treatment.

### 2.5. Tumor Necrosis Factor-Induced Prostate Cancer Stem Cell Killing Is Caused by Blunted NF-κB Activation

**Figure 8:**
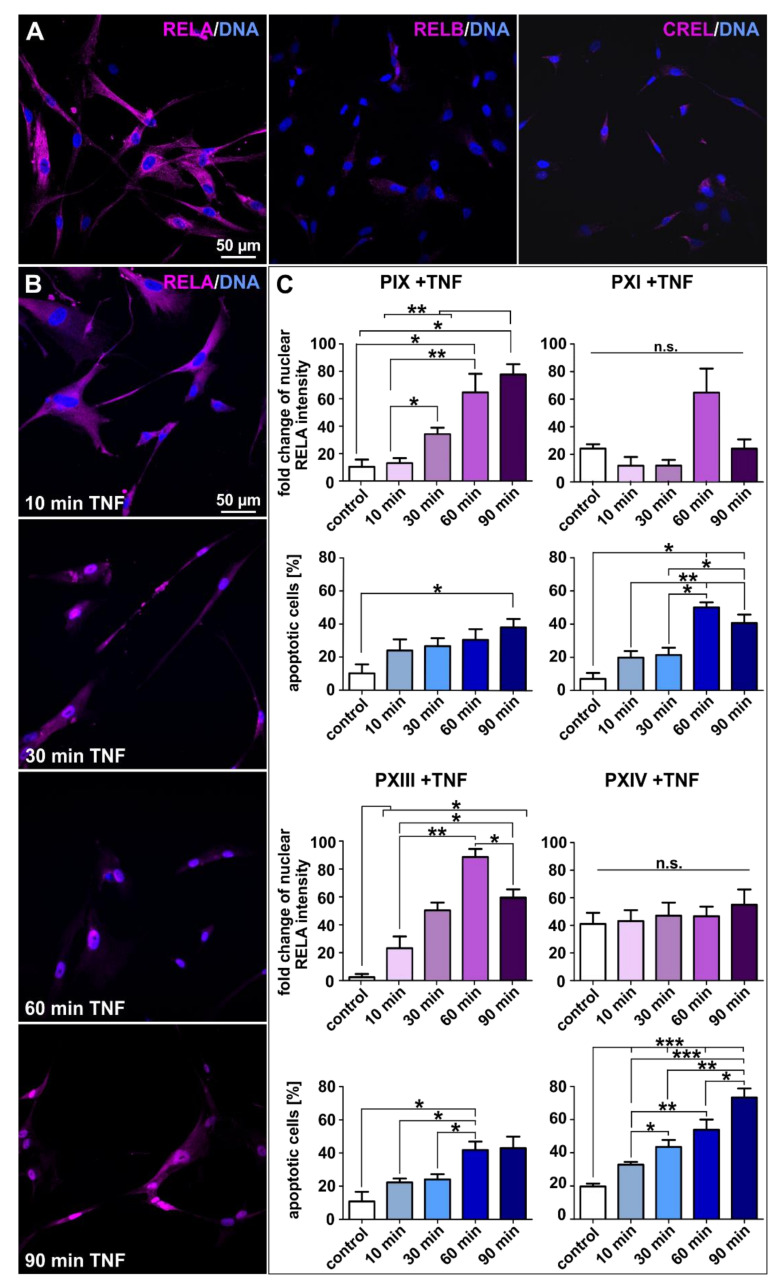
Tumor necrosis factor treatment led to profound cytotoxicity in prostate cancer stem cells unable to activate NF-κB. (**A**) The exemplary immunostaining of cultured PXIV prostate cancer stem cells (PCSCs) resulted in predominant NF-κB RELA expressions as well as slightly detectable RELB and CREL proteins expressions. (**B**) Exemplary images of immunocytochemically NF-κB RELA-stained PXIII PCSCs after treatment with tumor necrosis factor alpha (TNFα) for 10, 30, 60 and 90 min. The expressions of RELA in PCSCs seemed to shift from the cytoplasm to nucleus over the experimental duration. Nuclear counterstaining was performed using DAPI. (**C**) TNFα stimulations were evaluated by measuring the fold changes in nuclear RELA intensities and average percentages of apoptotic cells via quantification with ImageJ software (NIH, Bethesda, MD, USA). The PIX and PXIII PCSC populations displayed statistically significant upregulated fold changes of nuclear RELA intensities upon TNFα treatment (left panel, magenta graphs). The DAPI staining of nuclei displayed statistically high upregulations of apoptotic cells in PXI and PXIV PCSCs after treatment with TNFα (right panel, blue graphs). Statistical analysis was performed using Mann–Whitney U tests (minimum 50 cells per condition and population); * *p* ≤ 0.05, ** *p* ≤ 0.01 and *** *p* ≤ 0.001 were considered significant. Mean ± standard error of the mean (SEM).

As mentioned above, the formation and progression of cancers are associated with NF-κB signaling and deregulation [50,51], whereas activation is commonly reported to correlate with the initiation of cancer cell survival [52]. To gain detailed insights into PCSCs NF-κB signaling, we analyzed NF-κB subunit expressions before and after treatment with TNFα (Figure 8). The initial immunocytochemical staining for natural expressions of respective NF-κB subunits displayed the overall predominance of the cytoplasmically expressed RELA protein in each analyzed population (Figure 8A and Figure S11). All populations except PXIII showed slight expressions of RELB (middle panel Figure 8A and Figure S11). The expression of CREL was observed in all PCSC populations, but to a lesser extent than RELA (right panel Figure 8A and Figure S11), suggesting RELA as the most suitable NF-κB subunit for further TNFα-based induction experiments (Figure 8B,C). As aforementioned, the activation of NF-κB through pro-inflammatory cytokines such as TNFα has already been described in the literature [35,36,61]. In detail, we treated pre-cultured PCSC populations for 10, 30, 60 and 90 min with TNFα, and analyzed the potential differences between treated and control cells via immunocytochemistry. Firstly, the potential induction of NF-κB was visualized via the fluorescent localization of the RELA protein (exemplary images for PXIII in Figure 8B, and PIX, PXI and PXIV PCSCs in Figure S12) given that the simultaneous regulation of nuclear RELA translocation and overall NF-κB activation in cancer cells has already been reported [62]. Secondly, PCSC survival was examined via the condensation and fragmentation of DAPI-stained nuclei within the same images. Treated PCSCs shifted their RELA from the cytoplasm to nucleus, and stained nuclei manifested a condensed shape. We evaluated any further effects statistically (Figure 8C). Here, the high-level upregulation of the nuclear RELA protein was apparent in the PIX and PXIII PCSC populations, with a maximum of >80-fold increased intensity (left panels, magenta graphs). Surprisingly, the PCSCs of PXI and PXIV displayed no significant shift in RELA localization, and manifested no induction of the NF-κB transcription factor (right panels). In addition to RELA protein signals, cell death upon TNFα treatment was determined by measuring apoptotic cells (Figure 8C, blue graphs). Here, all analyzed PCSC populations displayed a statistically significant increase in levels of apoptotic cells following treatment with TNFα. However, the population-based analysis revealed dual effects. Whereas the PCSC populations PIX and PXIII, which showed strong NF-κB activation, contained a maximum of only 40% apoptotic cells (Figure 8C, left panels), PXI (Gleason score 3 + 4) and PXIV (Gleason score 4 + 5) PCSCs, which displayed blunted NF-κB activation, displayed high-level inductions of up to 70% apoptotic cells after TNFα treatment (Figure 8C, right panels). Consequently, blunted NF-κB activation resulted in robust PCSC killing efficacy following treatment with TNFα. The Gleason score of the PCa tissue from which the PCSCs originated seems to have no correlation with the potency of NF-κB activation, since the results for PXI and PXIV (3 + 4 and 4 + 5) were the opposite of those of PIX and PXIII (4 + 3 and 3 + 3).

## 3. Discussion

We successfully isolated PCSC populations from four parental tumor samples. We confirmed expressions of the specific CSC surface markers CD44, CD133 and *EPCAM*, as well as the CSC-associated transcription factors *SLUG, SNAIL, TWIST* and MYC. While CD133 is commonly related to stemness properties, it might also lead to tumorigenesis, metastasis and chemoresistance in CSCs (reviewed in [63]). The interaction of CD44 with histone-associated genes upregulated NF-κB activity and chemoresistance in breast tumor cells [64]. Interestingly, only PXIV PCSCs (Gleason score 4 + 5) showed expressions of the metastasis-associated gene *CXCR4*, indicating a potential correlation with the Gleason score. However, *EPCAM* was expressed in each population, and in a PCa model system, this is associated with EMT, and might contribute to metastatic plasticity [25]. *SLUG, SNAIL* and *TWIST*, besides being crucial to stem cell characteristics, correlate with an EMT fate in CSCs. In this regard, Bob Weinberg and co-workers have shown that the overexpression of SLUG in mammary epithelial cells results in EMT to CSCs [28]. The presence of TWIST was additionally reported to be a key regulator of embryonic morphogenesis and contributed directly to metastasis via promoting EMT in mammary carcinoma cells [26]. Moreover, the induction of distinct EMT programs via SNAIL and SNAIL family members, such as SLUG, has been outlined in normal mammary stem cells, as well as in tumor-initiating cells [27]. Thus, we can conclude that our isolated, spindle-shaped, mesenchymal-like cancer cells are PCSCs. Of note, we previously described ubiquitous expressions of CD44 and MYC in various CSCs from four different cancer types (glioblastoma multiforme, endometrioid, lung and prostate), including three of the PCSC populations we used here, which further verifies the stemness of these primary isolated PCSCs [29].

Here, we have shown that CSCs possess various mechanisms to evade cell death induced by clinical treatment. One outstanding example is the chemoresistance potential of CSCs, which we can examine via their high ALDH function (another possible marker for PCSCs) [24]. Li et al. identified, that *ALDH1A1*-expression in malignant PCSC populations was associated with the decelerated progression of disease. In contrast, Yu and co-workers described high ALDH activity as favorable for metastatic and tumorigenic cells but not so important for PCa cells with stem-like properties [65]. In our study, we detected individual fractions of PCSCs with high ALDH activity, strongly indicating their potential for metastasis and chemoresistance. In addition to chemoresistance, the invasion of the immune system in order to avoid the patients’ immunosurveillance is another CSC-mediated survival strategy. The presence of an immunoglobulin superfamily member, PD-1, was identified as active in the regulation of programmed cell death by Ishida and colleagues in 1992 [66]. PD-1 is expressed on various immune cells, such as B-cells, T-cells and NK cells, which are inter alia responsible for specific cancer cell lysis. Nevertheless, cancer cells’ inherent stemness characteristics directly suppressed the immune system by expressing the receptor ligands and surface markers PD-L1 and -L2 [32,33], which bind to the PD-1 receptor on immune cells, therefore impair the immune cells’ proliferation and thus evading the immune response. Consequently, we analyzed PD-L expressions at the protein level via flow cytometry and detected high quantities of both ligands. We further identified an increase in PD-L1/-L2^+^ cells after IFNγ treatment. These findings agree with recent literature, but here, different cancer-supportive side effects (such as the resulting PD-L induction in CSCs) were observed, along with the presence of pro-inflammatory cytokines, such as TNFα and IFNγ [34,35,36,37]. To examine the direct interaction of our isolated PCSCs with patient-protective immune cells, we co-cultured primary cells with enriched NK cells and analyzed PCSC lysis. With a maximum specific cancer cell killing of 9.75%, we detected no effects of cytotoxic NK cells on our PD-L1/-L2-expressing PCSCs (see Figure 5, left panels). Addressing this, a multicenter study of Hansen and colleagues identified that treatment with the PD-1 inhibitor pembrolizumab resulted in favorable side effect profiles and anti-tumor activities in a patient cohort with advanced PD-L1-positive PCa [67]. Therefore, we partially treated co-cultured PCSCs and NK cells with pembrolizumab, but we did not observe any enhancement in specific cancer cell lysis. In this context, Moretta et al. and Di Tomaso et al. described the potential inactivation of NK cell lysis as a result of MHC class I member expression [59,68]. In accordance with the literature, we examined the expression of MHC class I-related B2M and HLA-A2 in our isolated PCSCs, which were partially treated with IFNγ, and observed high expression levels via immunochemistry and flow cytometry. This could be explained by the “missing self-hypothesis”, suggesting that the inhibitory signals overpower NK cell-activating signals, and thus inhibit cytotoxicity [38,39,40]. B2M expression was strongly upregulated during IFNγ treatment, but even though the number of apoptosis-related marker CASP-3 stained cells increased, this did not lead to efficient PCSC killing.

In 1989, Alain Israel and co-workers stimulated the expression of mouse MHC class I genes via TNFα and revealed MHC class I molecules to be direct target genes of NF-κB [69]. We investigated the specific interplay of NF-κB with our isolated and NK cell-resistant PCSCs. We previously showed the specific GO-term enrichment of “NF-κB binding” in a broad CSC transcriptomic analysis [29]. Within the same study, we detected the ubiquitous presence of the respective NF-κB target genes *HLA-A* and *-B* after RNA sequencing; here, we also verified HLA-A2 expression on the protein level. In the present study, which is exclusively focused on CSCs originating from PCa, we determined the predominant expression of the NF-κB subunit RELA, and the lesser expressions of RELB and CREL. Even though the respective proteins were localized within the cytoplasm of the PCSCs, after treatment with the common NF-κB-inducer TNFα, two of the populations displayed nuclear RELA, suggesting robust transcription factor activation, as expected. To our surprise, PXI (Gleason score 3 + 4) and PXIV (Gleason score 4 + 5) showed no NF-κB induction but demonstrated significantly increased levels of apoptotic cells, which subsequently coincided with TNFα-induced cell death. In this light, many studies have postulated the relation between TNFα-induced NF-κB activation and the expression of chemoresistance and metastatic genes in solid cancers, such as PCa; however, none have shown direct signaling [53,54,55]. Furthermore, treatment with various chemotherapeutic agents, such as doxorubicin, 5-fluorouracil, cisplatin and paclitaxel, resulted in increased inflammation and the expression of TNFα (reviewed in [70]). We therefore conclude that chemotherapy might fail when patients’ PCSCs respond with activated NF-κB. In addition, the suppression of NF-κB activity resulted in increased apoptosis and chemosensitivity in colorectal CSCs and breast cancer cells, while reducing the specific malignancy of colorectal CSCs [71,72,73]. Moreover, an additional study by Joseph et al. reviewed the release of endogenous TNFα as a potential immunotherapeutic in general cancer [74]. Conclusively, after a detailed analysis of three pathways for the potential killing of CSCs from PCa, only one mechanism resulted in effective PCSC death independently of the Gleason score. Our findings reveal the central role of NF-κB RELA in the efficient TNFα-induced killing of PCSCs with blunted NF-κB activation. Regarding the optimization of PCa patient-specific treatment options after surgery, the culturing and PCSC analysis of NF-κB induction potency may improve supportive therapies, such as TNF-inducing chemotherapeutics and/or NF-κB inhibitors. 

Limitations: In the present study, each patient underwent a radical prostatectomy after PCa diagnosis. As listed in Section 4.1, follow-ups at a maximum of 25 months showed no evidence of disease before the submission of this manuscript. However, the biological recurrence of PCa and the further progression of metastases were reported to appear after a mean time of 42 months post-surgery [75]. Thus, studying patient-specific treatments after initial tumor removal may be beneficial to future PCa therapy and long-lasting patient survival.

## 4. Materials and Methods

### 4.1. Patients Clinical Characterizations

Prior to surgical prostate removal, initial PSA quantity was assessed in whole blood samples of all the patients, resulting in 0.5 ng/mL (PXIII tissue sample) to 25.0 ng/mL (sample PXI). All cases of PCa were diagnosed via medical examination, and each patient signed an informed consent form, approved by the ethics commission of the University of Münster (Germany) and the General Medical Council at Münster (Germany; approval reference number 2017-522-f-S), before surgery. During histopathological analyses, detailed TNM classifications (TNM) and subsequent Gleason scores were determined for malignant tumors (Table 2 and see also Table 1). Further adjuvant therapeutic strategies, such as androgen ablation or radiation therapy, were partially implemented concomitantly. Several months after surgery, donor-specific follow-ups were carried out, and the current health status of each patient was examined. Appropriative and detailed information of each donor’s clinical classifications is listed below in Table 2.

### 4.2. Isolation and Cultivation of Primary Prostate Cancer Stem Cell Populations

Cancer tissue samples were kindly provided by the Forschungsverbund BioMedizin Bielefeld/OWL (FBMB e.V.), at the Protestant Hospital of Bethel Foundation (Bielefeld, Germany) during histopathological analysis but prior to fixation of the resected organ at the Department of Pathology of the Protestant Hospital of Bethel Foundation. In detail, received tissues were macroscopically analyzed, small amounts of potential cancer nodules were sampled via biopsy punch (6 mm; pfm medical ag, Cologne, Germany) and stored in sterile phosphate-buffered saline (PBS; Life Technologies, Darmstadt, Germany) at 4 °C, until transporting to the cell culture lab. Simultaneously to the tissue sample carriage, the pathology analyzed the resected organ via frozen section to ensure the presence of a cancerous disease. After the existence of a PCa case was verified, cancer samples were used to isolate primary PCSCs, firstly by mechanical disintegration, then by enzymatic digestion with collagenase A (AOF, Worthington, Lakewood, CO, USA) and calcium chloride (300 µM; Sigma-Aldrich, Taufkirchen, Germany) for 2 h at 37 °C, while shaking. Tissue suspensions were centrifuged and cultured in a humidified incubator at 37 °C and 5% CO_2_ in growth media specific to CSCs containing Dulbecco’s modified Eagle’s medium/F-12 (Life Technologies), L-glutamine (2 mM; PAA Laboratories, Linz, Austria), penicillin/streptomycin (100 µg/mL; PAA Laboratories) and B-27 (1:50; Gibco, Grand Island, NY, USA). The growth factors bFGF-2 (40 ng/mL; Sigma-Aldrich) and EGF (20 ng/mL; Sigma-Aldrich) were additionally supplemented. Culture flasks were pre-coated with 0.1% gelatin (bovine skin type B; Sigma-Aldrich) in PBS, and 10% FCS (VWR, Darmstadt, Germany) was subsequently added to the media for adherent cultivation. As is well-known, the quantity of human tumorigenic PCSCs is defined as only approximately 0.1% of prostate cells within the tumor building [20]. Therefore, specific enrichment of PCSC populations was performed as previously described by Witte and Hertel et al. [29], using protocols for the serial trypsin treatment of CSCs, as reported by Walia et al. and Morata-Tarifa et al. [76,77]. Adherently grown cells from primary explant cultures (passage 0) were washed with PBS, treated for 5 min with trypsin (Sigma-Aldrich), and the subsequently detached cells were reseeded into new gelatin pre-coated culture flasks. The trypsinization procedure was repeated at least three times every 48 to 72 h to achieve adherently cultured and mesenchymal-shaped prostate carcinoma cells with specific stemness properties.

### 4.3. Culture of Free-Floating Spheres

As one potential indicator of stemness, previously enriched PCSC populations were seeded with 5 µg/mL heparin (Sigma-Aldrich) in growth media under serum-free conditions. After several days of culture, images of the formed spheres were taken via light microscopy (EVOS XL; Thermo Fisher Scientific, Waltham, MA, USA).

### 4.4. Immunochemistry

To immunohistochemically stain PCa slices, paraffin-embedded sections were first deparaffinized and rehydrated—PCa slices were washed with xylol (Sigma-Aldrich) for 20 min, followed by 10 min with ethanol absolute solution, and then they were hydrated in 90%, 80% and 70% ethanol, each for 5 min. For reconditioning of the epitope, the PCa sections were boiled for 20 min in 0.01 M citrate buffer (pH 6.0, lab-made) and chilled for 30 min at room temperature. The slides were washed in PBS with 0.02% Triton-X 100 (Sigma-Aldrich) for 10 min. The blocking of free binding sides and permeabilization were performed via incubation with 0.02% Triton-X 100, 10% appropriative serum (DIANOVA, Hamburg, Germany) and 1% bovine serum albumin (Sigma-Aldrich) in PBS for at least 2 h. After this, the following primary antibodies were diluted in blocking solution and applied for 1 h: anti-CD133 (1:100; NB120-16518; NovusBio, Bio-Techne, Wiesbaden-Nordenstadt, Germany) and anti-CD44 (1:50; 156-3C11; Cell Signaling, Frankfurt am Main, Germany). PCa slices were washed three times with PBS followed by incubation with secondary fluorochrome-conjugated antibodies using Alexa Flour 555 dyes (1:300; goat anti-mouse and goat anti-rabbit; Life Technologies) for 1 h with no light. Subsequent incubation with 4′,6-diamidino-2-phenylindole (DAPI; 1 μg/mL; Sigma-Aldrich) was performed for 10 min to ensure nuclear counterstaining. Following this, fluorescence imaging was carried out with a confocal laser scanning microscope (LSM 780; Carl Zeiss, Jena, Germany), and images were analyzed via ImageJ software (NIH, Bethesda, MD, USA).

For the immunocytochemical staining, primary PCSCs were seeded on top of sterilized cover slips with 50,000 cells per 4 cm^2^ in 12-well plates, cultured with 1 mL growth medium for 48 h. Adherently grown cells were fixed for 10 min with 4% paraformaldehyde in PBS. Cellular permeabilization and the blocking of free binding sides were performed using PBT solution, containing 0.02% Triton-X-100 and 5% goat serum in PBS for 30 min. Three washing steps with PBS followed, and then incubation with primary antibodies for 1 h. The primary antibodies used for immunocytochemistry were anti-CD133 (1:100), anti-CD44 (1:400), anti-Nestin (1:200; MAB5326; Merck, Darmstadt, Germany), anti-MYC (10 μg/mL; Y69; Abcam), anti-N-MYC (2.5 μg/mL; NCM II 100; Abcam), anti-NF-κB p65 (RelA; 1:400; D14E12; Cell Signaling), anti-RelB (1:100; D7D7W; Cell Signaling), anti-cRel (1:100; Cell Signaling), anti-B2M (1:50; REAL845; Miltenyi Biotec, Bergisch Gladbach, Germany) and anti-CASP-3 (1:400; Asp175/5A1E; Cell Signaling). After further washing steps, secondary fluorochrome-conjugated antibodies (1:300; Alexa Flour 488 and −555 dye; goat anti-mouse or goat anti-rabbit; Life Technologies) were applied for 1 h, protected from light. Nuclear staining was performed in a similar fashion to immunohistochemistry, with the addition of another washing step with PBS. Stained PCSCs were embedded in Mowiol 4-88 (Carl Roth GmbH, Karlsruhe, Germany) upside down on microscope slides, and subsequently fluorescent-imaged via the confocal laser scanning microscope (LSM 780). Protein localizations were analyzed via ImageJ software.

### 4.5. RNA Isolation and Reverse-Transcription PCR

RNA from 1 × 10^6^ cultured and pooled PCSCs was isolated using the NucleoSpin^®^ RNA Plus kit (Macherey-Nagel, Düren, Germany) according to the manufacturer’s guidelines. The quality and concentration of isolated RNAs were assessed via nanodrop ultraviolet spectrophotometry. For each sample, 1 µg of RNA was transcribed into cDNA according to the protocol of Thermo Fisher Scientific. Analyses of PCSC gene expression profiles were performed with the taq-S PCR Kit (New England Biolabs, Frankfurt am Main, Germany) according to the manufacturer’s guidelines. The specific primer sequences used for characterization are listed in Table 3 below.

Amplified RT-PCR transcripts were loaded on agarose gel (Sigma-Aldrich) with 0.001% ethidium bromide (Carl Roth GmbH), run at 100 V, and imaged with a trans-illuminator (UVsolo TS; Biometra, Göttingen, Germany).

### 4.6. Examination of the Population Doubling Time

To analyze the proliferative behavior of PCSCs, specific growth rates and respective population doubling times were determined. For this, 300,000 cells per 9 cm^2^ were seeded in 6-well plates and cultured for 4 h as the internal control after initial PCSC attachment, and for 72 h to calculate the final quantity of cells. PCSCs were harvested, counted, and the growth rates and population doubling times were calculated using the formulas below.

(1) $$Growth\;rate=\frac{\ln\left(x_{t}\right)-\ln\left(x_{0}\right)}{t-t_{0}}$$

(2) $$Population\;doubling\;time=\frac{\ln\left(2\right)}{growth\;rate}$$

### 4.7. Flow Cytometry

For the determination of ALDH activity, as well as PD-L1/-L2, B2M and HLA-A expressions, the PCSC populations were cultured in growth medium and then partially pre-treated with the cytokine IFNγ (10 ng/mL; Peprotech, Hamburg, Germany) for 72 h. PCSCs were detached via trypsin, harvested, and analyzed via flow cytometry on a Gallios Flow Cytometer (Beckman Coulter Life Sciences, Krefeld, Germany). The following antibodies were used for flow cytometry: anti-CD274 PE (PD-L1; 1:50; MIH1; BD Biosciences, Heidelberg, Germany), anti-CD273 APC-Vio^®^770 (PD-L2; 1:10; MIH18; Miltenyi Biotec), anti-B2M PE (1:50; REAL845; Miltenyi Biotec) and anti-HLA-A2 PE (1:50; BB7.2; BD Biosciences), according to manufacturer’s instructions. IsoIgG1-PE (1:10; IS5-21F5; Miltenyi Biotec) and REA Control APC-Vio^®^770 (1:10; REA293; Miltenyi Biotec) were used as isotype control antibodies. The measurement of ALDH activity was performed using the ALDEFLUOR™ Kit from STEMCELL Technologies (Vancouver, BC, Canada), in accordance with their guidelines and the approach of Moreb et al. [78]. For all experiments, dead cells were excluded with Propidium Iodide (PI; 1µg/mL; Thermo Fisher Scientific), and automatic compensation and the resulting analyses were conducted using the evaluation software Kaluza 1.0 from Beckman Coulter Life Sciences.

### 4.8. Co-Culture with Natural Killer Cells

NK cells from primary buffy coats (kindly provided by the Institut für Laboratoriums und Transfusionsmedizin, HDZ NRW, Bad Oeynhausen, Germany) were enriched via an adherence-based protocol previously described by Selvan and Dowling et al. [79]. PBMCs were isolated via density gradient centrifugation with Lymphoprep™ (STEMCELL Technologies) and NK cells were enriched using the RosetteSep™ human NK cell enrichment cocktail (STEMCELL Technologies) according to the manufacturer’s instructions. The NK cells were cultured in NK-MACS^®^ medium (Miltenyi Biotec) containing 5% AB serum (via calcification of blood plasma; kindly provided by the Institut für Laboratoriums- und Transfusionsmedizin, HDZ NRW), 100 U/mL penicillin and 10 µg/mL streptomycin (Sigma-Aldrich) and human Interleukin-2 (IL-2; 500 U/mL; Miltenyi Biotec), for 25 days in cell culture dishes. The NK cell medium was changed every 48 to 72 h until the cultured cells reached a confluence of 90–100%. Afterward, the cells were harvested using trypsin as described above, resuspended to a density of 0.5 × 10^6^ cells/mL medium, and reseeded in new culture flasks. For the NK cell-based cytotoxicity assays, adherently cultured PCSCs were pre-treated with IFNγ (10 ng/mL) for 72 h before harvesting. Target cells were stained with Cell Trace Violet (2.5 µM; Invitrogen, Carlsbad, CA, USA), and 200,000 PCSCs per 9 cm^2^ were seeded on 6-well plates in regular growth medium, partially supplemented with pembrolizumab (10 nM; Selleckchem, Houston, TX, USA). Previously isolated NK cells were also harvested and added to the cultured PCSC populations in 1 mL CSC medium (ratio: 2:1 and 10:1), partially containing pembrolizumab. After 4 h of incubation, the co-cultured cells were detached, resuspended in autoMACS^®^ rinsing solution (Miltenyi Biotec) with 2% FCS and PI, and subsequently analyzed on a Beckman Coulter Gallios Flow Cytometer with the evaluation software Kaluza 1.0 from Beckman Coulter Life Sciences. Specific NK cell-directed PCSC lysis was determined using the following formula:

(3) $$Cell\;lysis=\frac{\left(viable\;cells_{control}-viable\;cells_{co–cultured}\right)}{viable\;cells_{control}}\times 100\%$$

### 4.9. Stimulation of the Major Histocompatibility Complex Class I

To analyze the relation between PCSC populations and the immune system, cellular stimulation with IFNγ was performed and assessed via immunocytochemistry, as described above. Here, the successful activation of major histocompatibility complex I (MHC I) member B2M and apoptosis-related protein CASP-3 were detected after 24 and 48 h of stimulation with IFNγ (10 ng/mL). For each condition, at least five images were analyzed, and the respective fold changes in cellular B2M intensity were assessed via the following formula:

(4) $$Fold\;change\;=\;\frac{\left(F_{treatment}-F_{min}\right)}{\left(F_{max}-F_{min}\right)}\times 100$$

The average percentages of CASP-3-positive cells were measured via imaging and analyzing the immunocytochemical stainings. For both these evaluations, the statistical quantifications were based on at least five exemplary images per condition.

### 4.10. NF-κB Transcription Factor Activation

To stimulate the NF-κB transcription factor, PCSC populations were initially seeded in 24-well plates on sterilized cover slips, as described above. After 48 h of culture, the medium was refreshed and supplemented with the cytokine TNFα (10 ng/µL; Merck, Germany). The cells were stimulated for 10, 30, 60 and 90 min to analyze the effects on the nuclear RELA protein in PCSCs. Fixation and immunofluorescent staining was performed as explained above. For each condition, at least five exemplary images were analyzed, and respective fold changes in nuclear RELA intensity were assessed via Equation (4). The average percentage of apoptotic cells was evaluated by analyzing the nuclear condensation and the size of the DAPI stained nuclei, and heavily condensed nuclei and/or nuclei < 50 µm^2^ were designated as positively apoptotic.

### 4.11. Statistical Analysis and Figure Design

Data were analyzed using the Prism V5.01 software (GraphPad Software, Inc., San Diego, CA, USA). Normality was tested via the D’Agnostino and Pearson omnibus normality test. Non-parametric one-way ANOVA (Kruskal–Wallis test) with Dunn’s multiple comparison and Mann-Whitney U tests, or a paired *t*-test, were performed to assess differences between multiple groups. For each analysis, *p* ≤ 0.05 was considered statistically significant. The evaluated data are displayed as the means ± standard error of the mean (SEM). All figures were designed with the CorelDRAW Graphics Suite 2018 software (Corel Corporation, Ottawa, ON, Canada).

## Figures and Tables

**Table 1:** Donor classification of prostate cancer samples.

Donor ID	Therapy	Tumor Typing and Characterization	Gleason Score	Age
PIX	RPx	Acinar adenocarcinoma	4 + 3	71
PXI	RPx	Acinar adenocarcinoma	3 + 4	57
PXIII	CPx	Muscle-invasive, infiltrating urothelial carcinoma of the bladder (high grade) and acinar adenocarcinoma	3 + 3	83
PXIV	RPx	Locally advanced acinar adenocarcinoma	4 + 5	72

**Table 2:** Donors’ clinical characterizations and therapy information.

ID	Initial PSA (ng/mL)	TNM Classification	Adjuvant Therapy	Follow-Up (Months)	Current Status
PIX	15.7	pT2c pN1 R0	No	25	No evidence of disease (ned)
PXI	25.0	pT3a pN0 R1	Androgen ablation	24	ned
PXIII	0.5	pT2c pN0 R0 (PCa) and pT3a pN0 R0 (BCa)	No	23	ned
PXIV	6.5	pT4a pN0 R1	Radiation	23	ned

**Table 3:** Primer sequences used for gene amplification via reverse-transcription polymerase chain reactions.

Target Gene	Sequence (5′-3′)	Size (bp)
*ACTB*	GAGAAGATGACCCAGATCATGT	333
	CATCTCTTGCTCGAAGTCCAG	
*AR*	GACATGCGTTTGGAGACTGC	471
	GTTGTTGTCGTGTCCAGCAC	
*CXCR4*	ATGGCAAGAGACCCACACAC	85
	ATATTGGGCGGGAGTGTCAG	
*EPCAM*	GCTGGCCGTAAACTGCTTTG	100
	ACATTTGGCAGCCAGCTTTG	
*NKX3-1*	ACAAGATGCACTCGCTGTGA	132
	CTAGCAGCAGTGTGGAGACC	
*SLUG*	TCGGACCCACACATTACCTT	125
	TTGGAGCAGTTTTTGCACTG	
*SNAIL*	CCCAATCGGAAGCCTAACTA	157
	GGACAGAGTCCCAGATGAGC	
*TWIST*	GTCCGCAGTCTTACGAGGAG	159
	CCAGCTTGAGGGTCTGAATC	

## Data Availability

The data presented in this study are available in the article and the Supplementary Materials.

## Supplementary Materials

The following are available online at https://www.mdpi.com/article/10.3390/ijms22168901/s1, Figure S1: Potential sphere formation as well as CD44/CD133 and nestin immunostainings of cultured PIX, PXIII and PXIV PCSC populations, Figure S2: MYC and N-MYC immunocytochemistry of PXI, PXIII and PXIV PCSC populations, Figure S3: Full-size RT-PCR images of analyzed target gene expressions in PCSC populations, Figure S4: DEAB controls of flow cytometric-analyzed ALDH expressions, Figure S5: Dot plots including gating strategies of flow cytometric-analyzed PD-L1 expressions, Figure S6: Dot plots including gating strategies of flow cytometric-analyzed PD-L2 expressions, Figure S7: Exemplary B2M immunostainings of IFNγ-treated PIX, PXIII and PXIV PCSC populations, Figure S8: Exemplary CASP-3 immunostainings of IFNγ-treated PIX, PXIII and PXIV PCSC populations, Figure S9: Dot plots including gating strategies of flow cytometric-analyzed B2M expressions, Figure S10: Dot plots including gating strategies of flow cytometric-analyzed HLA-A2 expressions, Figure S11: Immunocytochemistry of NF-κB subunit expressions in primary PIX, PXI and PXIII PCSCs, Figure S12: Exemplary RELA immunostainings of TNFα-treated PIX, PXI and PXIV PCSC populations, Table S1: Mean fluorescence intensities of PD-L1 and -L2 after flow cytometric analysis, Table S2: Mean fluorescence intensities of B2M and HLA-A2 after flow cytometric analysis.
