# Supplementary material for: Analysis of Several Pathways for Efficient Killing of Prostate Cancer Stem Cells: A Central Role of NF-κB RELA
Source: Int J Mol Sci. 2021 Aug 18;22(16):8901. doi: 10.3390/ijms22168901 (PMC8396252; doi:10.3390/ijms22168901)
Supplement: Supplementary file 1 [file ijms-22-08901-s001.zip › ijms-1273889-supplementary.pdf]

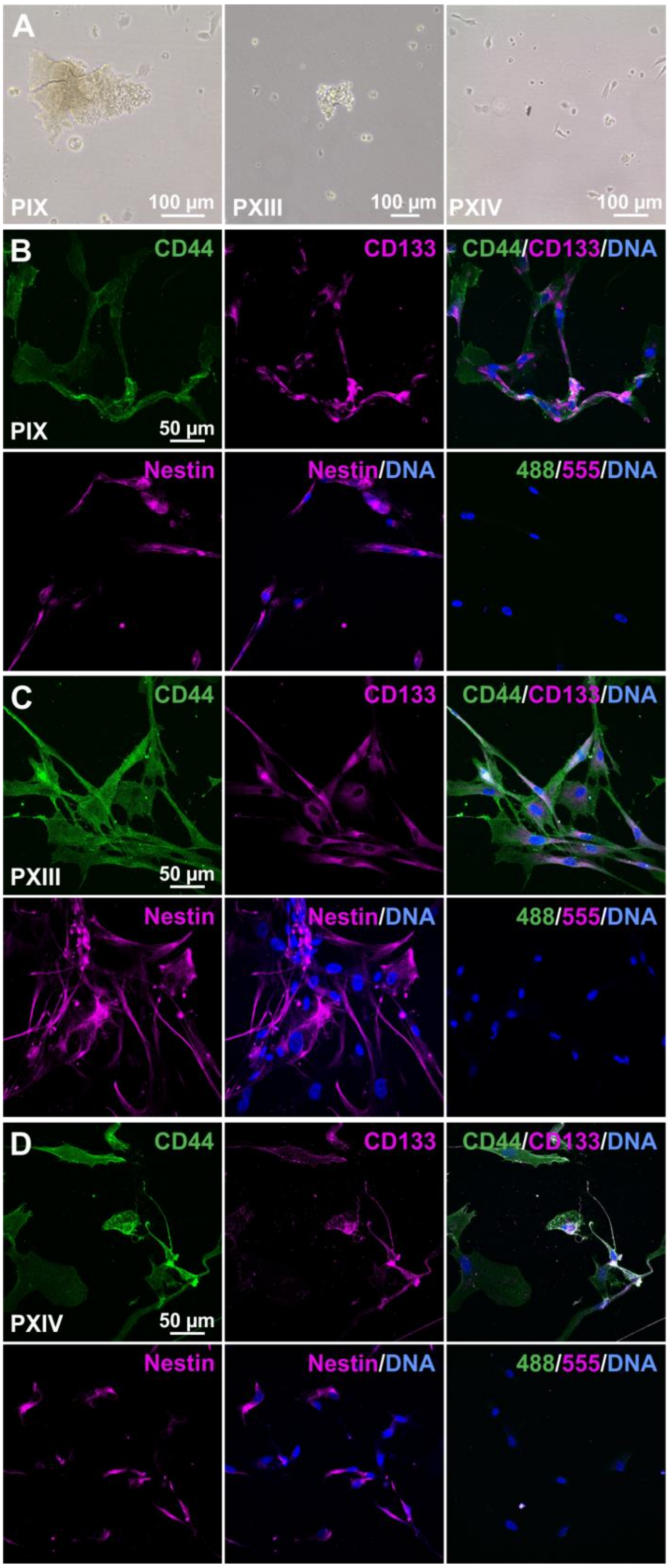

Figure S1: Potential sphere formation as well as CD44/CD133 and Nestin immunostainings of cultured PIX, PXIII and PXIV PCSC populations.

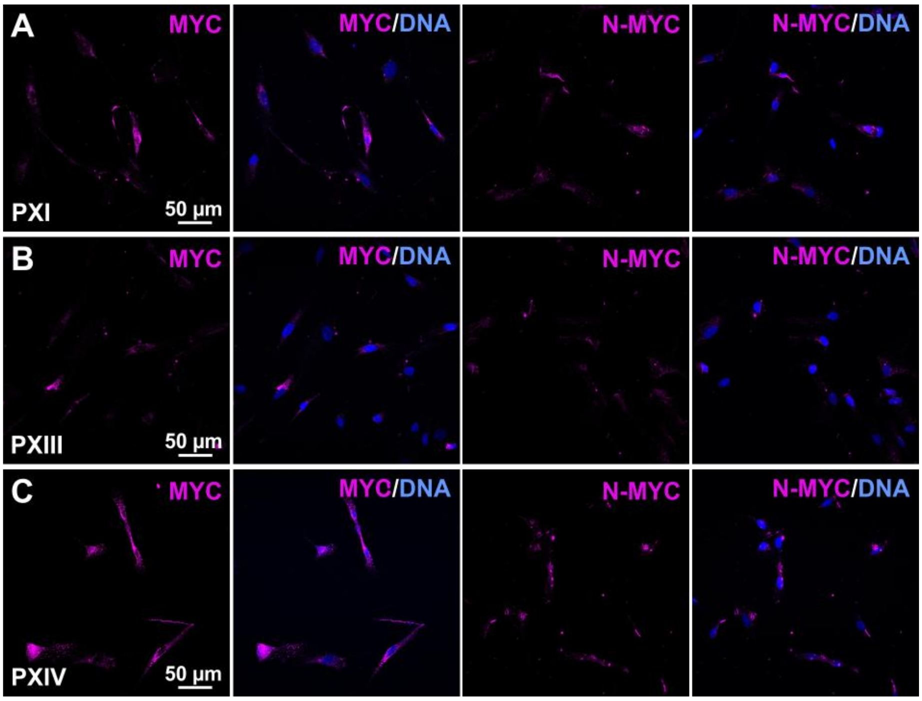

Figure S2: MYC and N-MYC immunocytochemistry of PXI, PXIII and PXIV PCSC populations.

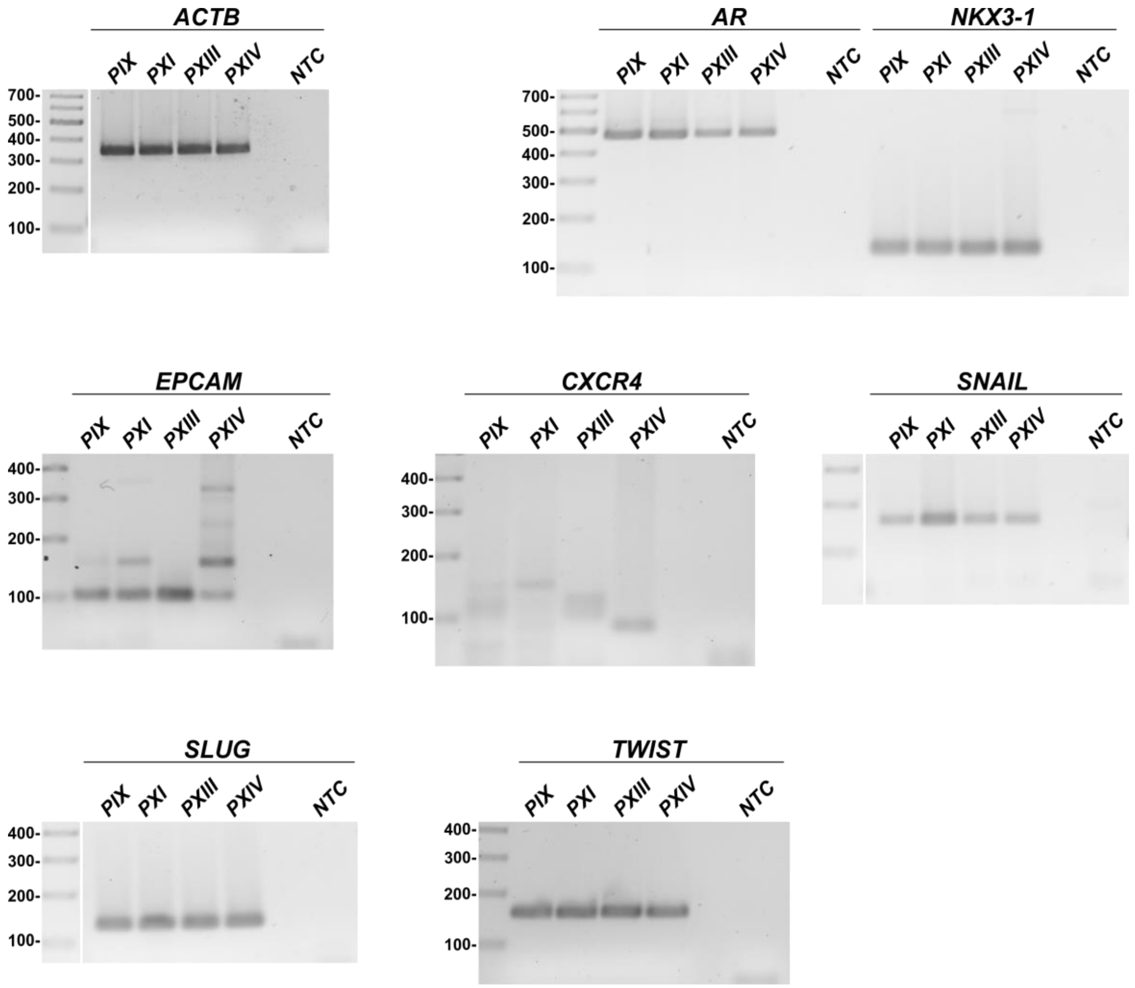

Figure S3: Full size RT-PCR images of analyzed target gene expressions in PCSC populations.

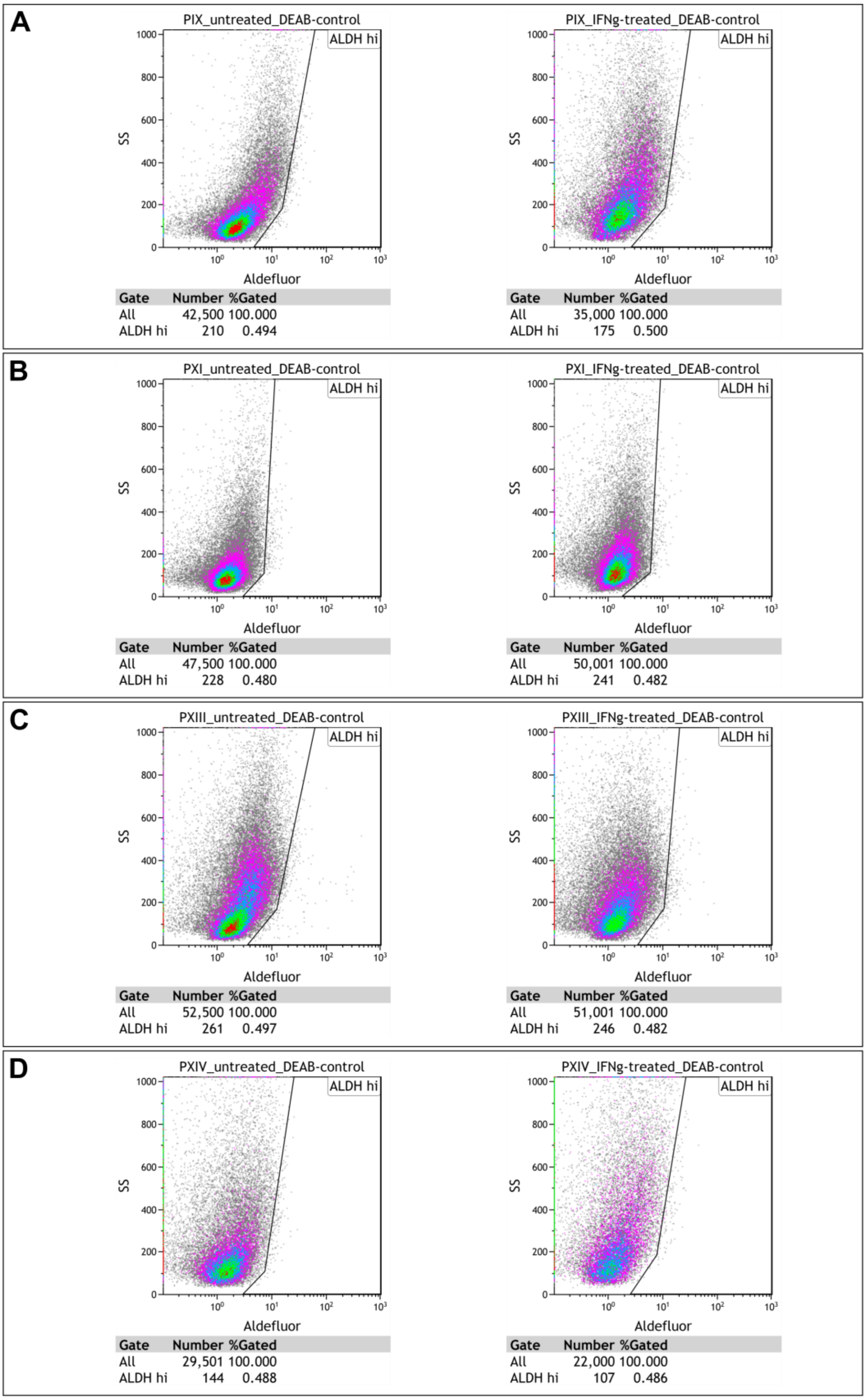

Figure S4: DEAB controls of flow cytometric-analyzed ALDH expressions.

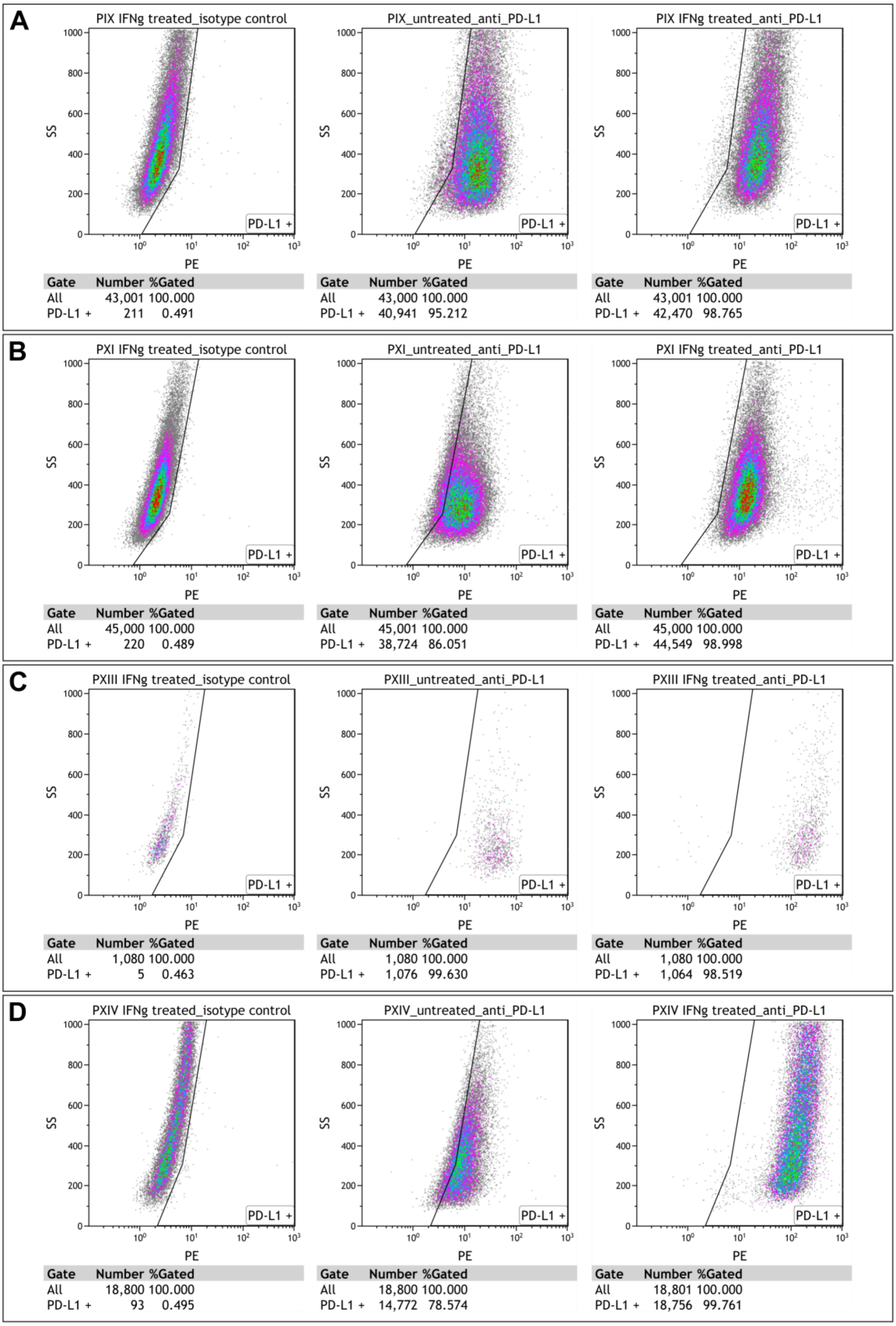

Figure S5: Dot plots including gating strategies of flow cytometric-analyzed PD-L1 expressions.

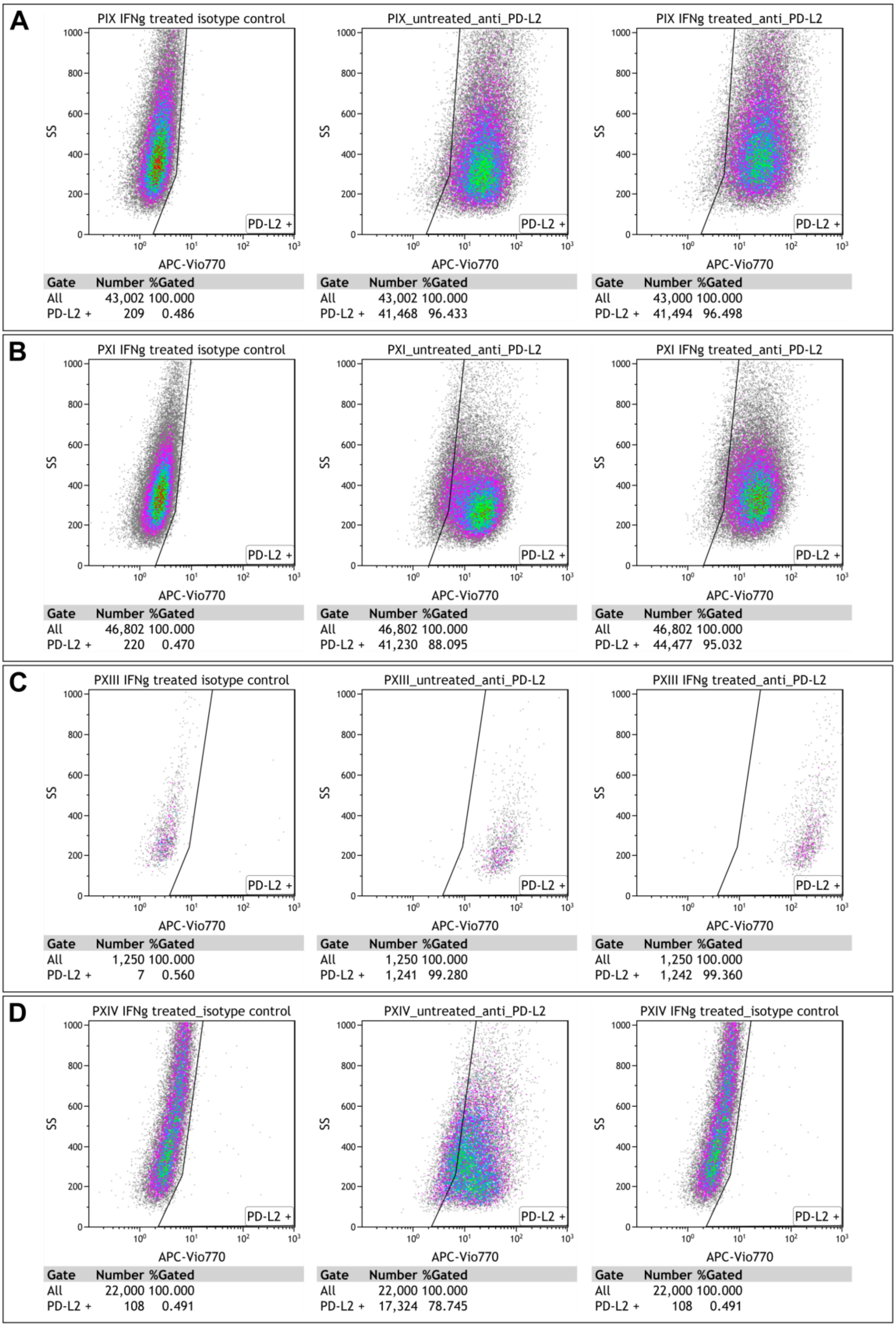

Figure S6: Dot plots including gating strategies of flow cytometric-analyzed PD-L2 expressions.

Table S1: Mean fluorescence intensities of PD-L1 and –L2 after flow cytometric analysis.

Sample ID	Condition	Mean fluorescence intensity (MFI) PD-L1	MFI PD-L2
PIX	control	21,649	29,275
	IFNγ pre-treated	28,364	35,591
PXI	control	10,172	20,110
	IFNγ pre-treated	18,206	24,397
PXIII	control	44,854	62,699
	IFNγ pre-treated	223,453	249,716
PXIV	control	11,384	20,841
	IFNγ pre-treated	153,728	104,884

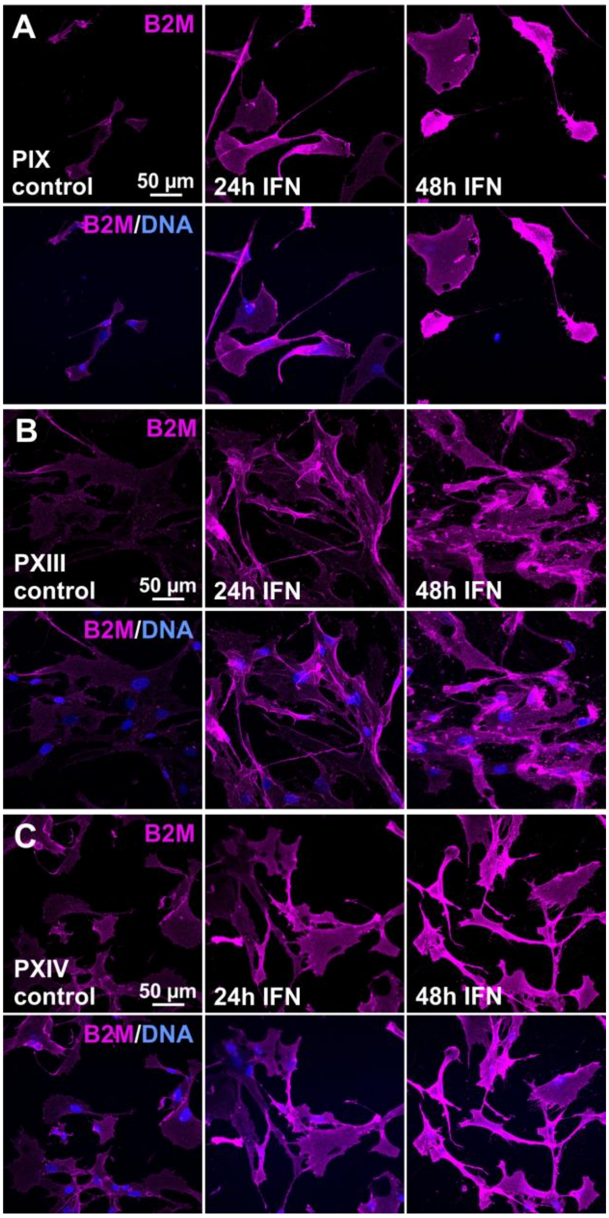

Figure S7: Exemplary B2M immunostainings of IFNγ-treated PIX, PXIII and PXIV PCSC populations.

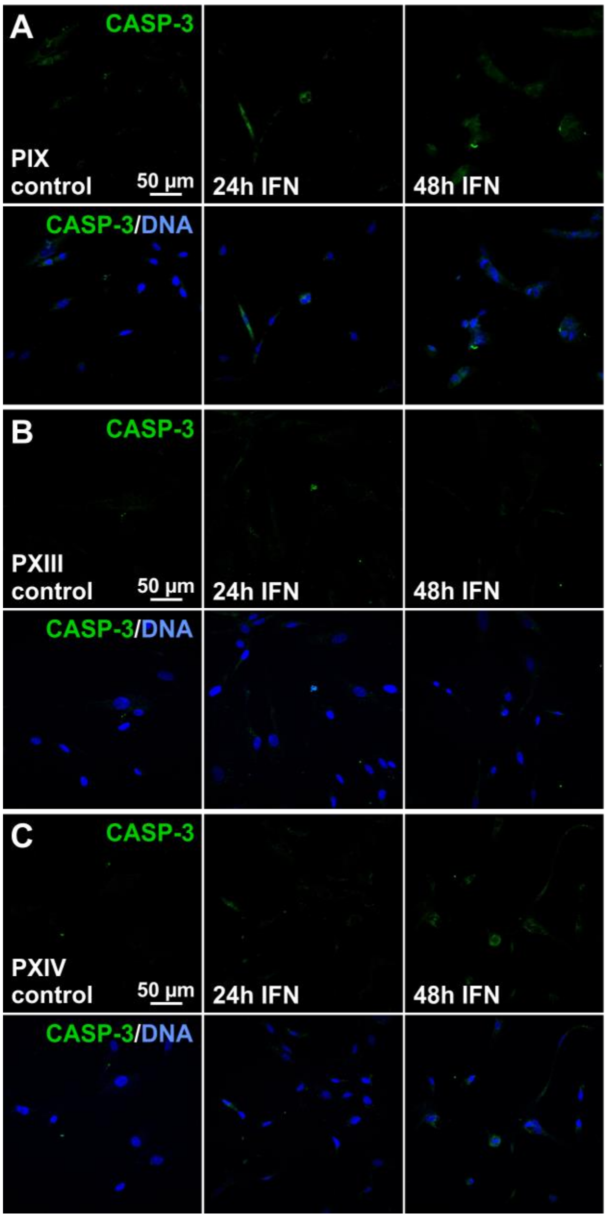

Figure S8: Exemplary CASP-3 immunostainings of IFNγ-treated PIX, PXIII and PXIV PCSC populations.

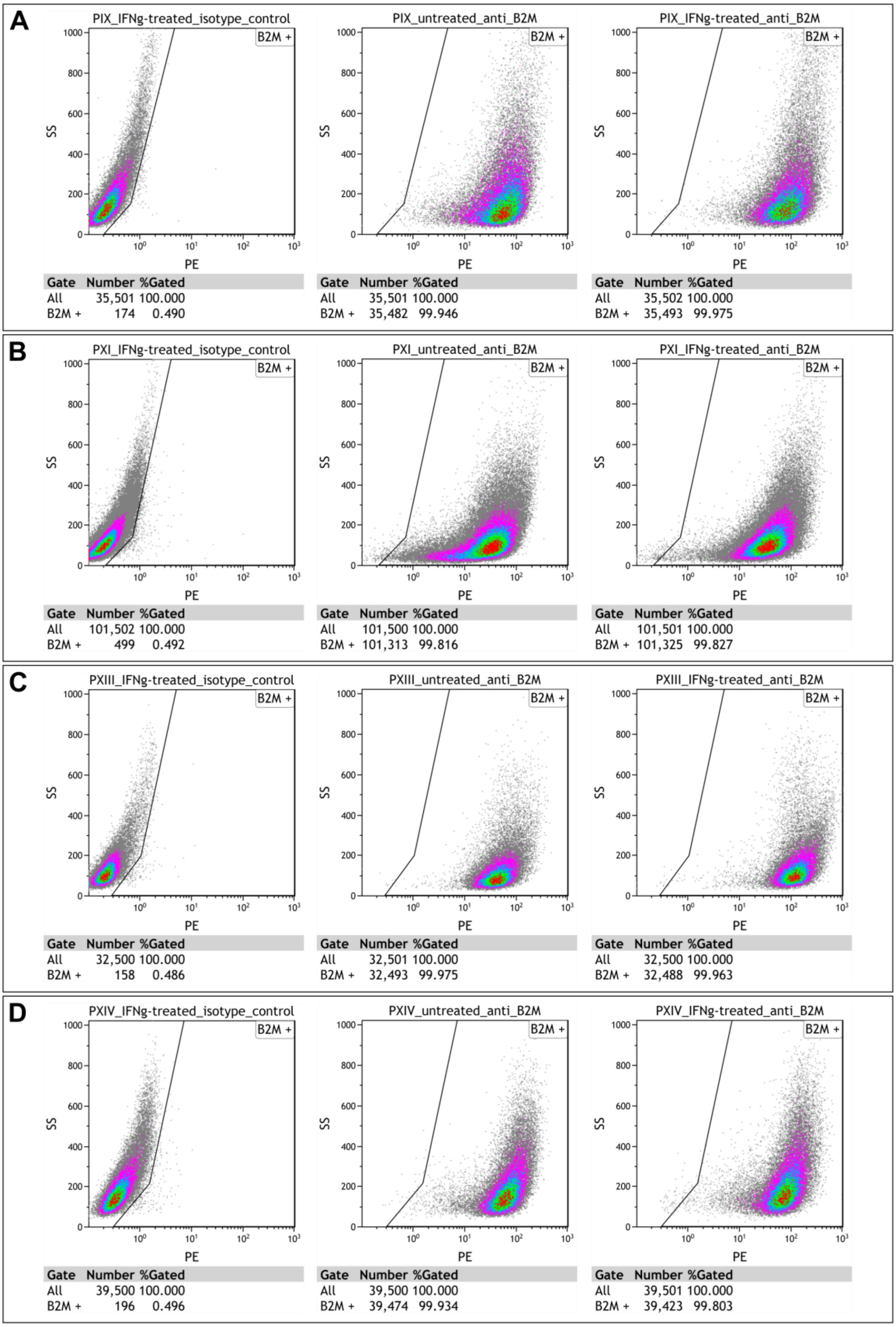

Figure S9: Dot plots including gating strategies of flow cytometric-analyzed B2M expressions.

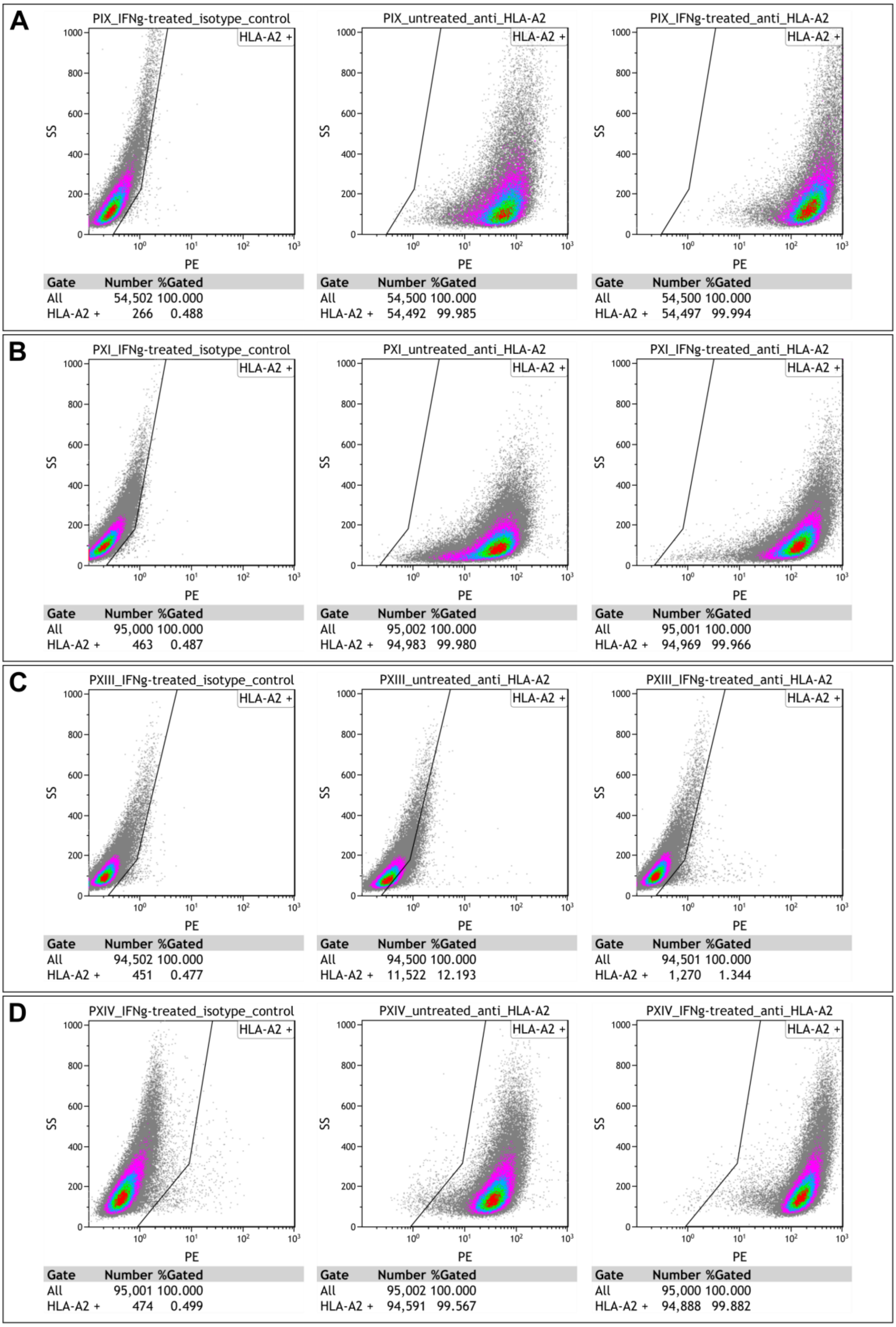

Figure S10: Dot plots including gating strategies of flow cytometric-analyzed HLA-A2 expressions.

Table S2: Mean fluorescence intensities of B2M and HLA-A2 after flow cytometric analysis.

Sample ID	Condition	Mean fluorescence intensity (MFI) B2M	MFI HLA-A2
PIX	control	67,444	70,235
	IFNγ pre-treated	108,696	296,777
PXI	control	37,822	46,91
	IFNγ pre-treated	50,267	174,073
PXIII	control	54,421	0,451
	IFNγ pre-treated	146,833	0,336
PXIV	control	77,459	46,265
	IFNγ pre-treated	93,594	202,444

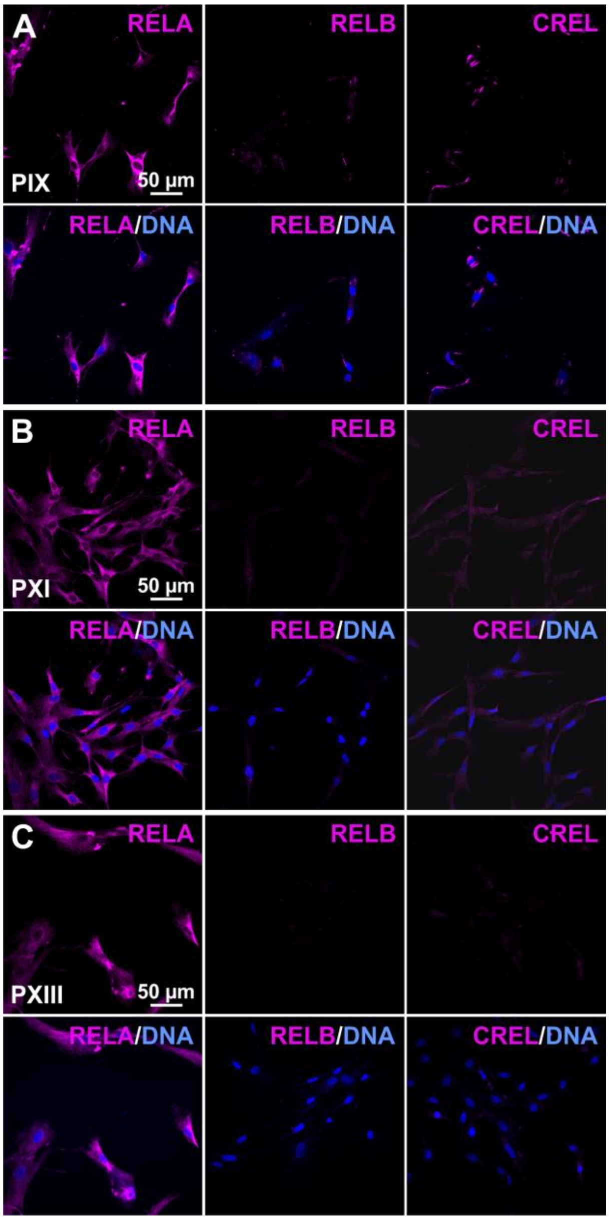

Figure S11: Immunocytochemistry of NF-κB subunit expressions in primary PIX, PXI and PXIII PCSCs.

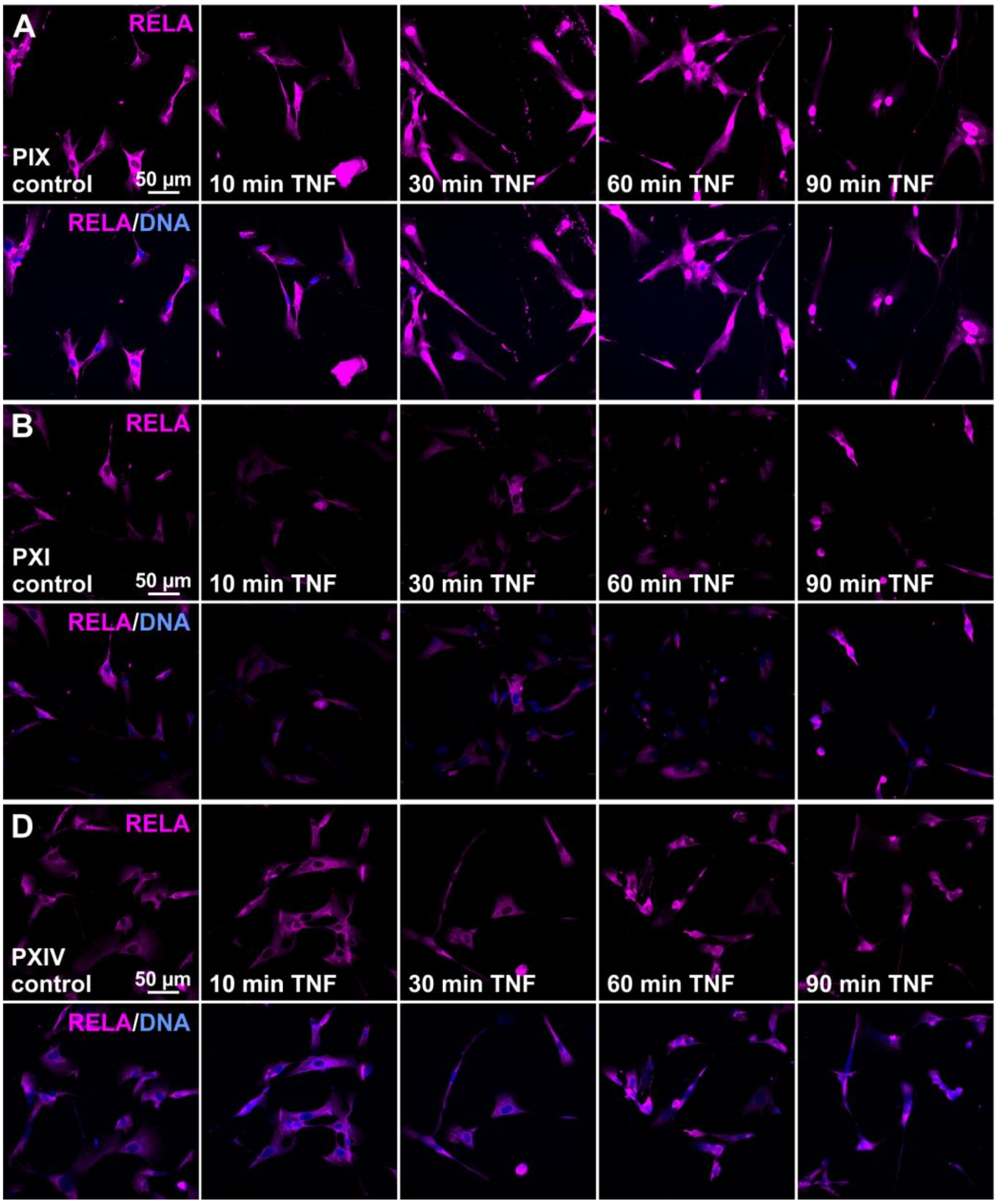

Figure S12: Exemplary RELA immunostainings of TNFα-treated PIX, PXI and PXIV PCSC populations.
